# Co-regulation of HIV control and cytomegalovirus pp65-specific IL-1β and TNF-α responses by genetic variants in the MHC region

**DOI:** 10.1371/journal.ppat.1014355

**Published:** 2026-07-14

**Authors:** Suzanne D. E. Ruijten, Jéssica C. dos Santos, Victoria Rios-Vazquez, Adriana Navas, Hanneke Maas, Albert L. Groenendijk, Marc J. T. Blaauw, Louise E. van Eekeren, Wilhelm A. J. W. Vos, Rainer Knoll, Manoj Kumar Gupta, Rob ter Horst, Javier Botey-Bataller, Nienke van Unen, Yang Li, Anna C. Aschenbrenner, Joachim L. Schultze, Cheng-Jian Xu, Mihai G. Netea, Andre J. A. M. van der Ven, Vasiliki Matzaraki

**Affiliations:** 1 Department of Internal Medicine and Infectious Diseases, Radboudumc, Nijmegen, The Netherlands; 2 Department of Internal Medicine and Department of Medical Microbiology and Infectious Diseases, ErasmusMC, Erasmus University, Rotterdam, The Netherlands; 3 Systems Medicine, Deutsches Zentrum für Neurodegenerative Erkrankungen (DZNE), Bonn, Germany; 4 Centre for Individualised Infection Medicine (CiiM), a joint venture between the Helmholtz Centre for Infection Research (HZI) and the Hannover Medical School (MHH), Hannover, Germany; 5 TWINCORE, a joint venture between the Helmholtz-Centre for Infection Research (HZI) and the Hannover Medical School (MHH), Hannover, Germany; 6 CeMM Research Center for Molecular Medicine of the Austrian Academy of Sciences, Vienna, Austria; 7 Cluster of Excellence Resolving Infection Susceptibility (RESIST; EXC 2155), Hannover Medical School, Hannover, Germany; 8 Lower Saxony Center for Artificial Intelligence and Causal Methods in Medicine (CAIMed), Hannover, Germany; 9 Genomics and Immunoregulation, Life and Medical Sciences Institute, University of Bonn, Bonn, Germany; 10 Department of Immunology and Metabolism, Life and Medical Sciences Institute, University of Bonn, Bonn, Germany; University of Duisburg-Essen: Universitat Duisburg-Essen, GERMANY

## Abstract

The spontaneous control of HIV infection in the absence of antiretroviral therapy, termed HIV control, is associated with genetic variation in the Major Histocompatibility Complex (MHC) locus. These variants are known to influence the immune response to HIV itself. However, people living with HIV are often co-infected with other pathogens that can also elicit immune responses, which might also be regulated by these variants. Here, we assessed whether genetic variants associated with HIV control influence cytokine responses to various co-pathogens. HIV-control-associated single nucleotide polymorphisms (SNPs) were enriched among variants regulating TNF-α and IL-1β production upon CMV pp65 peptide pool stimulation. The top enriched SNPs, rs1128175-A and rs2853971-A, were linked to lower odds of HIV control and increased cytokine responses to CMV. These SNPs were in linkage disequilibrium (LD) with classical HLA alleles HLA-B*07:02 and HLA-C*07:02. Intracellular cytokine staining showed CMV serostatus-dependent production of TNF-α by monocytes and CD8 T cells. The rs1128175-A/rs2853971-A/HLA-B*07:02/HLA-C*07:02 haplotype was associated with increased IFN-γ production by CD8 T cells upon CMV pp65 peptide pool stimulation, indicating an effect on memory responses. Quantitative trait locus (QTL) mapping showed that rs1128175 and rs2853971 influence *HLA-B* and *HLA-C* expression, DNA methylation levels and cell-type-specific cis-effects on chromatin accessibility, as well as CD8 T cell subset abundance. These QTL associations suggest that variants associated with poor HIV control are linked to heightened pro-inflammatory responses to CMV pp65 through effects on antigen presentation, epigenetic modifications, gene expression and immune cell repertoire, potentially negatively affecting HIV control status.

## Author summary

HIV controllers are individuals that can spontaneously control an HIV infection without treatment. Understanding how their immune system controls the infection could help to develop new therapies for HIV. It is known that genetics plays an important role in this ability, but how genetic variants affect the production of immune mediators to HIV itself and common co-infections is yet to be understood. Here, we show that genetic variants associated with poorer HIV control are associated with increased release of pro-inflammatory cytokines in response to cytomegalovirus, a virus that people living with HIV are commonly co-infected with. These genetic variants are located within the MHC region, which is important for antigen presentation, as part of the adaptive immune system. We found that the cytokines are mainly produced by CD8 T cells and monocytes. Additionally, we show that these genetic variants influence the expression of genes involved in immune responses, possibly by epigenetic modifications, and the composition of immune cells in the blood. Together, our findings show that genetic variants associated with HIV control upregulate the immune response to CMV through multiple mechanisms, suggesting that CMV-specific immune responses might be associated with HIV control.

## Introduction

Only a minority of people living with HIV (PLHIV) can exert long-term control of the virus in the absence of combined antiretroviral therapy (cART) [1–3]. These individuals, known as HIV controllers (HICs), are able to maintain minimal plasma viral loads, have CD4 counts within the normal range without cART treatment, do not develop clinical disease, and are less likely to transmit HIV [4]. HICs are classified as either elite controllers (ECs) or viremic controllers (VCs), depending on plasma viral loads [5]. Independent of the HIV control status, over 80% people living with HIV are co-infected with cytomegalovirus (CMV) [6,7]. CMV has a profound impact on the immune system, especially on the T cell and NK cell repertoire, and is known to induce memory responses upon antigenic restimulation, characterized by increased IFN-γ production [8–11]. Moreover, CMV-HIV co-infection has been suggested to contribute to persistent inflammation in PLHIV under suppressive cART, resulting in increased prevalence of co-morbidities and increased HIV persistence in PLHIV [8,12].

Both HIV control and the interindividual variation in pro-inflammatory response to pathogens are known to be influenced by genetic and non-genetic factors. Previous genetic studies have shown the protective effect of genetic variants on HIV control, namely mutations in the *CCR5* locus, including a 32 bp deletion in *CCR5* (CCR5delta32), and single nucleotide polymorphisms (SNPs) in the Major Histocompatibility Complex (MHC) locus. These SNPs are often in strong linkage disequilibrium (LD) with classical Human Leukocyte Antigen (HLA) alleles, most notably in HLA-B and HLA-C [13–21]. The genetic associations with the MHC class I locus point to an important role for CD8 T cell-mediated immunity in HIV control [22–25]. In line with these findings, studies in macaques have shown increased SIV replication upon CD8 T cell depletion [26–28]. Besides the role of HIV-specific CD8 T cells, recent studies provided evidence for an important role of innate immune responses in HIV control [29]. In addition, single nucleotide polymorphisms (SNPs) impact *ex vivo* cytokine production capacity to various stimuli in PLHIV, underscoring the role of host genetics in modulating cytokines responses, including those induced by CMV [30]. However, the mechanisms regulating these pro-inflammatory responses to CMV in HIV controllers are currently unknown.

In this study, we explored the relationship between HIV control and cytokine responses to CMV and other pathogen-derived ligands. Given the importance of genetics for the HIV controller phenotype and *ex-vivo* cytokine production, we first investigated whether genetic variants associated with HIV control influence *ex vivo* cytokine production in response to various (non)-microbial stimuli, including CMV, in PLHIV from the 2000HIV cohort. Our findings reveal genetic co-regulation of HIV control and CMV pp65-specific IL-1β and TNF responses driven by genetic variants in the MHC region. Furthermore, through a functional genomics approach, we demonstrated that these genetic variants associate with epigenetic marks and gene expression in the MHC locus, as well as circulating immune cell composition, which provided insights on the mechanism by which MHC variants contribute to HIV control.

## Results

### SNPs associated with HIV-control are enriched in variants that influence pro-inflammatory cytokines responses to CMV pp65

**Fig 1:**
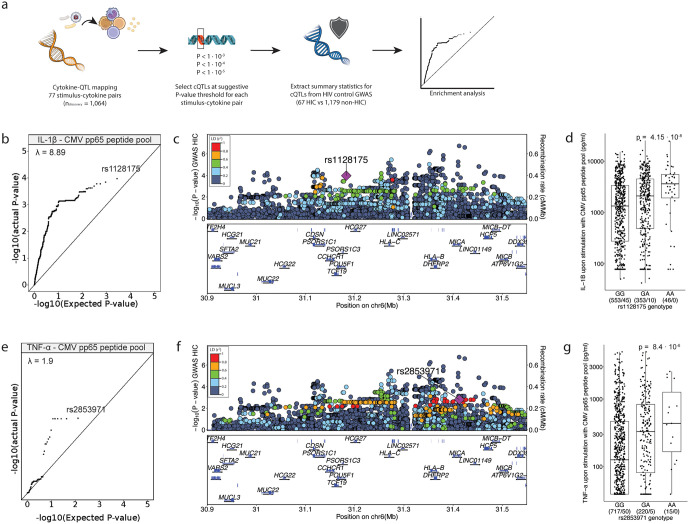
HIV control SNPs are enriched in cytokine-QTLs for IL-1β and TNF-α production upon stimulation with a CMV pp65 peptide pool. **(a)** Schematic overview of the enrichment analysis. First, summary statistics from the GWAS of HIV control were extracted for suggestive cQTLs at three different P-value thresholds across all stimulus-cytokine pairs. Enrichment analysis was then performed to assess whether HIV-control associated SNPs influence the ex vivo cytokine production **(b and e)** Enrichment of HIV control SNPs in cQTLs at P < 1 ⋅ 10^-5^ for IL-1β **(b)** and TNF-α **(e)** production upon stimulation with a CMV pp65 peptide pool. The x-axis represents the expected -log_10_(P-value) under a uniform distribution, the y-axis represents the actual -log_10_(P-value) from the GWAS on HIV control. The lambda inflation value is shown on the top left **(c and f):** Regional association plot for HIV control of top enriched SNPs rs1128175 **(c)** and rs2853971 **(f)**. The x-axis represents the chromosomal position, the upper plot shows the -log_10_(P-value) from the GWAS on HIV control, with colors representing linkage disequilibrium (LD) with the top enriched SNP. The lower part of the plot shows all genes in the respective genetic region. Boxplots of IL-1β **(d)** and TNF-α **(g)** production upon stimulation with a CMV pp65 peptide pool, stratified based on the rs1128175 **(d)** or rs2853971 **(g)** genotype. On the x-axis are the SNP genotypes and their frequencies (total count/count in HIC) and the y-axis shows cytokine concentration. P-values represent the P-values of associations with the cytokine production identified in the discovery cohort.

To assess whether SNPs associated with HIV control influence the *ex vivo* cytokine production, we tested for enrichment of HIV-control SNPs among cytokine quantitative trait loci (cQTLs), defined as SNPs associated with variation in *ex vivo* cytokine response. For this, we overlaid the summary statistics of the genome-wide association study (GWAS) for HIV control from our cohort with cQTL summary statistics previously mapped in the same cohort [30,31] (Figs 1a, S1).

The enrichment analysis was performed using cQTLs at three different suggestive P-value thresholds to prevent bias arising from the choice of a single P-value cutoff (P < 1 ∙ 10^-3^, P < 1 ∙ 10^-4^, P < 1 ∙ 10^-5^). We identified a consistent enrichment only in variants that influence IL-1β and TNF-α production upon 24 hour stimulation with a CMV pp65 peptide pool (Figs 1b, 1e, S2, S3 and S4).

At *P* < 1 ⋅ 10^-5^, the top enriched SNPs were rs1128175 and rs2853971, which are in moderate linkage disequilibrium (r^2^ = 0.38, D’ = 0.86) within the MHC region, regulating IL-1β and TNF-α production respectively (Fig 1b-1g). Specifically, rs1128175-A, linked to reduced odds for HIV control (P_GWAS_ = 1.1 ∙ 10^-4^, OR_GWAS_ = 0.30), was associated with increased IL-1β production upon stimulation with a CMV pp65 peptide pool (P_QTL_ = 4.15 ∙ 10^-8^, β_QTL_ = 0.28) (Fig 1d). Its association with HIV control was confirmed at genome-wide significance in individuals of European ancestry in the International HIV controller study (IHCS), a large-scale GWAS of HIV control, including over 500 HICs (P = 6.5 ∙ 10^-15^, β = -0.886) and its association with IL-1β production to CMV pp65 was confirmed in the validation cohort of the 2000HIV study (P_QTL_ = 4.4 ⋅ 10^-4^, β = 0.38) [13]. Similarly, rs2853971-A was associated with lower odds for HIC (P_GWAS_ = 1.6 ∙ 10^-3^, OR_GWAS_ = 0.23) and increased TNF-α production upon stimulation with a CMV pp65 peptide pool (P_QTL_ = 8.4 ∙ 10^-6^, β_QTL_ = 0.32) (Fig 1g). While the summary statistics of this SNP itself were not reported in the IHCS, a proxy (rs2523612-G, r^2^ = 0.82, OR = 0.31, P = 5.1 ∙ 10^-3^ in the 2000HIV study), showed a suggestive association in individuals of European ancestry in the IHCS (β = -0.688, P = 5.9 ⋅ 10^-7^) [13]. The association of SNP rs2853971-A with TNF-α production upon CMV pp65 peptide pool stimulation showed the same direction of effect in the validation cohort, but did not reach nominal significance (P_QTL_ = 0.097, β = 0.23). Given the effect of these SNPs on the response to CMV, we tested whether anti-CMV IgG levels influence the genetic associations with HIV control or CMV-specific cytokine production. We observed no significant interaction of anti-CMV IgG titres with HIV control (rs1128175: OR = 0.687, P = 0.24, rs2853971: OR = 0.579, P = 0.23), or *ex vivo* cytokine production (rs1128175: IL-1β CMV pp65 peptide pool: β = -0.041, p = 0.44, rs2853971: TNF-α CMV pp65 peptide pool: β = 0.033, p = 0.65).

Of note, rs1128175 is in moderate linkage disequilibrium with classical MHC class I alleles HLA-C*07:02 (r^2^ = 0.54, D’ = 0.98) and HLA-B*07:02 (r^2^ = 0.46, D’ = 0.95). r2853971 was not imputed in our MHC genetic dataset, but its proxy rs2596540 (r^2^ = 0.94, OR_GWAS_ = 0.246, P_GWAS_ = 2.4 ∙ 10^-3^) is in strong LD with HLA-C*07:02 (r^2^ = 0.80, D’ = 0.94) and HLA-B*07:02 (r^2^ = 0.95, D’ = 0.99). Given the strong association between HIV control and genetic variants in the MHC region, we assessed whether the observed enrichment was specific to cQTLs for IL-1β and TNF-α production upon CMV pp65 peptide pool stimulation, rather than variants with no cQTL effect within the MHC region. Using permutations with LD-independent SNPs matched by MAF to the cQTLs within two window sizes (< 500 kb or < 1Mb; see Methods), we generated a 95% confidence interval for the λ value based on random SNPs from the region. We observed that the λ value for enrichment of HIV-control SNPs in cQTLs for IL-1β production upon stimulation with a CMV pp65 peptide pool fell outside the 95% confidence interval using both windows, indicating specific enrichment (λ = 8.89, 95% CI_500kb_ = 3.99 – 4.73, 95% CI_1Mb_ = 2.86 – 3.6) (Methods; S5 Fig), However, for TNF-α, this was the case using a 500 kb (λ = 1.90, 95% CI = 0.66 – 1.88), but not a 1 Mb window (λ = 1.90, 95% CI = 0.73 – 2.12). This suggests that the enrichment is specific for cQTLs for IL-1β production upon stimulation with a CMV pp65 peptide pool, whereas the evidence for TNF-α for was less pronounced. Together, these results showed that SNPs associated with reduced odds for HIV control upregulate pro-inflammatory responses to CMV pp65. The LD between rs1128175 and rs2853971 and HLA alleles indicates that antigen presentation in the context of HLA-B*07:02 and HLA-C*07:02 may affect cytokine production upon stimulation with a CMV pp65 peptide pool.

### HIV-control associated SNPs regulating pro-inflammatory responses to CMV pp65 have pleiotropic effects

We next investigated whether these top enriched SNPs may influence the production of multiple cytokines in response to a CMV pp65 peptide pool or additional stimuli (pleiotropy). To test the presence of such pleiotropic effects, we extracted all possible associations for both SNPs using the summary statistics of cQTLs from the 2000HIV discovery and validation cohort. To account for multiple testing, we set a strict, Bonferroni-corrected threshold for testing two SNPs and 77 cytokine-stimuli pairs with an alpha-level of 0.05, resulting in a P-value threshold of 3.2 ⋅ 10^-4^ for the discovery cohort. SNP rs1128175, associated with IL-1β production upon stimulation with a CMV pp65 peptide pool, did not show any additional significant associations. SNP rs2853971-A was associated with increased IL-1β and MIP-1α upon stimulation with a CMV pp65 peptide pool, in addition to its association to TNF-α production upon CMV pp65 peptide pool stimulation (S1 Table, S6 Fig), suggesting that rs2853971 upregulates a broader pro-inflammatory response to CMV pp65.

### Monocytes and CD8 T cells are the primary producers of cytokines upon CMV re-exposure

**Fig 2:**
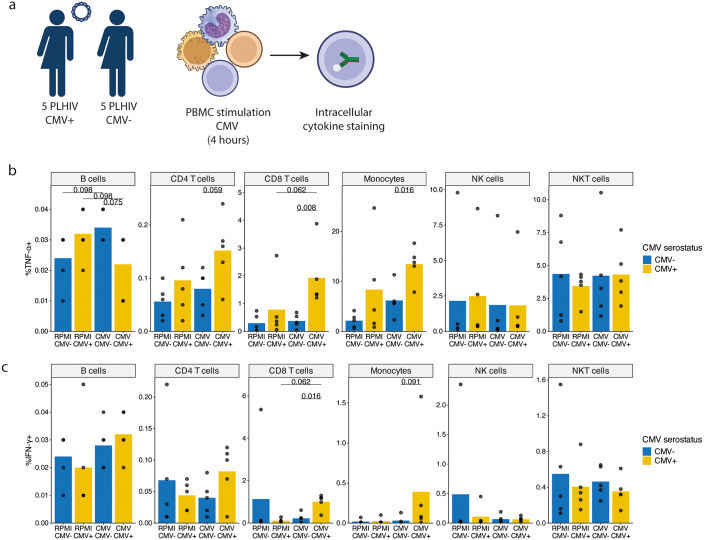
Intracellular expression of TNF-α and IFN-γ upon peripheral blood mononuclear cell (PBMC) stimulation with a CMV pp65 peptide pool in CMV+ and CMV- PLHIV. **(a)** Schematic overview of intracellular cytokine staining experiment. PBMCs from 10 PLHIV (5 CMV+ and 5 CMV-) were stimulated with RPMI (negative control) or a CMV pp65 peptide pool for 4 hours, of which 3 hours in the presence of brefeldin A. Intracellular cytokine expression in CMV+ and CMV- individuals were measured using flow cytometry in six major cell types: B cells (CD19+), CD4 T cells (CD3 + CD4 + CD8-), CD8 T cells (CD3 + CD4-CD8+), monocytes (CD14+), NK cells (CD3-CD56+), and NKT cells (CD3 + CD56 + CD8-) **(b + c)** Percentages of TNF-α+ and IFN-γ+ cells within each cell subset, respectively. Bar heights represent mean percentages, and color represents CMV serostatus; CMV- in blue and CMV+ in yellow. P-values from Wilcoxon signed-rank test (for comparisons within CMV serostatus groups) and Wilcoxon rank-sum test (for comparisons between CMV serostatus groups) are shown. Only P-values < 0.10 are shown.

We next aimed to determine which cell types produce TNF-α and IL-1β upon stimulation with a CMV pp65 peptide pool. For this, we assessed the percentages of monocytes, B cells, NK, NKT, CD4 and CD8 T cells expressing TNF-α and IL-1β intracellularly upon exposure to a CMV pp65 peptide pool, using RPMI as a negative control and LPS and PMA/ionocmycin as a positive control in 5 CMV seropositive and 5 CMV seronegative PLHIV (Fig 2a). HLA types of these individuals can be found in S2 Table. In addition to TNF-α and IL-1β, we measured intracellular production of IFN-γ, which is often used as a measurement of CMV memory responses [32–34].

Upon stimulation with a CMV pp65 peptide pool, increased percentages of monocytes expressing TNF-α were observed in CMV+ compared to CMV- PLHIV (P = 0.016) (Fig 2b). Such difference was not observed upon stimulation with the TLR4 agonist LPS (S7c Fig). Similarly, the expression of TNF-α by CD8 T cells was enhanced in CMV+ PLHIV compared to CMV- PLHIV (P = 0.008) upon CMV pp65 peptide pool stimulation. In addition, a similar trend was observed in CD4 T cells (P = 0.059) (Fig 2b). We did not observe changes in IL-1β expression upon CMV pp65 peptide pool exposure in any of the cell types studied (S8c Fig). The assessment of the IFN-γ expression in response to a CMV pp65 peptide pool further confirmed the increased responsiveness of CD8 T cells in CMV+ PLHIV (P = 0.016) (Fig 2c). PMA/Ionomycin showed a general increase in IFN-γ production in CMV+ compared to CMV- PLHIV in all cell types (S9c Fig). Of note, by comparing the immune cell composition of PLHIV based on their CMV serostatus, (CMV seropositive (CMV+) versus CMV seronegative (CMV-)), we observed a trend towards increased percentages of CD8 T cells (P = 0.056) in CMV+ PLHIV in comparison to CMV- PLHIV irrespective of the stimulation with the CMV pp65 peptide pool. No differences were observed for the remaining immune cells studied (S7b Fig). Together these results point to monocytes and CD8 T cells as the main cells responsible for TNF-α production upon stimulation with a CMV pp65 peptide pool. Moreover, the heightened responsiveness observed in CMV+ PLHIV upon re-exposure to CMV antigen further confirms the presence of a CMV memory responses induced in PLHIV.

### The rs1128175-A/rs2853971-A/HLA-B*07:02/HLA-C*07:02 haplotype is associated with increased CD8 T cell responses upon stimulation with a CMV pp65 peptide pool

**Fig 3:**
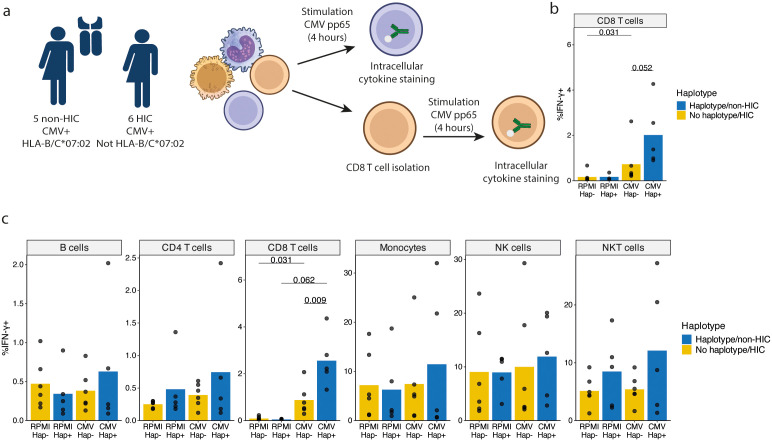
Intracellular cytokine staining of IFN-γ upon stimulation of PBMCs and the CD8 T cell-enriched PBMC fraction with a CMV pp65 peptide pool in PLHIV with and without the rs1128175-A/rs2853971-A/HLA-B*07:02/HLA-C*07:02 haplotype (a) Schematic overview of the intracellular cytokine staining experiment. 5 non-HICs carrying the rs1128175-A/rs2853971-A/HLA-B*07:02/HLA-C*07:02 haplotype (Hap+) and 6 HICs without the haplotype (Hap-) were selected. PBMCs and CD8 T cells enriched by positive selection were stimulated with RPMI or a CMV pp65 peptide pool for 4 hours, of which 3 in the presence of brefeldin A. IFN-γ expression was measured using flow cytometry in six major cell types: B cells (CD19+), CD4 T cells (CD3 + CD4 + CD8-), CD8 T cells (CD3 + CD4-CD8+), monocytes (CD14+), NK cells (CD3-CD56+), and NKT cells (CD3 + CD56 + CD8-) **(b)** Percentage of IFN-γ + CD8 T cells (y-axis) in the enriched CD8 T cell fraction stratified by stimulation and the presence of the haplotype (x-axis). For the RPMI condition in non-HICs with the haplotype, two samples were excluded due to low yield, resulting in a slightly lower sample size. **(c)** Percentage of IFN-γ+ cells within each cell type (y-axis) stratified by stimulation and the presence of the haplotype (x-axis). Colors represent the presence of the haplotype in the donor. Statistical testing was performed using a Wilcoxon signed-rank test for comparisons within haplotype groups and a Wilcoxon rank-sum test for comparison between haplotype groups. Only P-values < 0.10 are shown.

To test the effect of the genetic variants on intracellular cytokine production, we compared IFN-γ production to a CMV pp65 peptide pool between five non-HICs carrying the rs1128175-A/rs2853971-A/HLA-B*07:02/HLA-C*07:02 haplotype and six HICs, non-carriers of the haplotype (Fig 3a, S3 Table). Given that we measured a response to a peptide pool, we hypothesized that CD8 T cells recognize these peptides in the context of MHC class I to generate the primary response. To confirm the relevance of CD8 T cells, we added a condition in which we isolated CD8 T cells using positive selection.

Non-HICs carrying the haplotype showed increased IFN-γ production upon stimulation with a CMV pp65 peptide pool in CD8 T cells as part of the PBMC fraction (P = 0.009), but not in any of the other cell types (Fig 3c). This effect was also present in isolated CD8 T cells, although it did not reach statistical significance (P = 0.052; Fig 3b). In contrast to a CMV pp65 peptide pool, stimulation with an Epstein-Barr virus (EBV) BRLF1 peptide pool lead to a minimal IFN-γ production in CD8 T cells, and the IFN-γ response did not differ carriers and non-carriers of the risk haplotype (S10 Fig). This indicates that the haplotype associated with lower likelihood of HIV control was associated with increased IFN-γ production by CD8 T cells, specifically in response to a CMV pp65 peptide pool.

### SNPs associated with reduced odds for HIV control shape IL-1β and TNF-α response to CMV by regulating the expression of MHC genes through epigenetic changes

Next, we aimed to understand how these SNPs influence HIV control and the pro-inflammatory responses to CMV by investigating their functional effects using different approaches. First, we tested whether these SNPs influence the expression of nearby genes in a 1 Mb window in PBMCs from PLHIV. For this, we extracted *cis*-eQTL summary statistics from the 2000HIV cohort, which describe the association between the SNP genotype and the gene expression levels in *cis* [30]. The results demonstrated that rs1128175 influences the expression of six genes in the MHC locus at a suggestive P-value threshold (P_discovery_ < 0.01, P_validation_ < 0.05; Tables 1, S4). Amongst others, the rs1128175-A allele, associated with lower odds for HIV control and increased cytokine production to a CMV pp65 peptide pool, was associated with decreased *DDR1* expression and increased *HLA-C*, *TCF19* and *HLA-B* expression. The second SNP, rs2853971, had an eQTL effect for five genes, with the A allele associated with decreased *HCG27* and increased *HLA-B* and *DDAH2* expression (Tables 1, S4). The regulatory effect of rs1128175 and rs2853971 on the expression of certain genes was also found in QTL datasets from whole blood of healthy individuals (Tables 1, S5). Second, we evaluated whether genes within a 1 Mb window from the top SNPs were differentially expressed in PBMCs of HICs compared to non-HICs. Amongst others, we observed a reduced expression of *HLA-C* (log2 fold change = -0.052; nominal P = 0.02) in PBMCs of HICs compared to non-HICs, in accordance with the eQTL effect of rs1128175 (S6 Table).

**Table 1:** Prioritization of putative causal genes, epigenetic marks and cell types for top SNPs associated with HIV control and pro-inflammatory cytokine response upon stimulation with a CMV pp65 peptide pool. For mQTLs, only top features were prioritized. Full summary statistics of all QTLs can be found in S4, S5, S7–S9 Tables.

*rsID*	*Chr*	*Position*	*Risk Allele*	*GWAS HIC*		*Prioritized features*
				*P-value*	*OR*	
rs1128175	6	31182658	A	1.1 ∙ 10^-4^	0.3	*ATP6V1G2* (a), *C6orf15* (h), *CDSN* (d), *DDR1* (a)(b), ENSG000000272501 (a)(b), ENSG00000288813 (b), *HLA-B* (a)(c), *HLA-C* (a)(b)(c)(e)(h), *HLA-L* (b), *HLA-S* (b), *MICA* (d), *MICB* (g)*, MIR6891* (b), *PSORS1C1* (f), *PSORS1C3* (b), *TCF19* (a)(b), *XXbac-BPG181B23.7* (h), *XXbac-BPG299F13.14* (h), Neutrophils PD-L1+ (j), CD8 + Tc17 (j)
rs2853971	6	31412564	A	1.6 ∙ 10^-3^	0.23	*ATP6V1G2* (b), *C2* (g), *C4B* (b), *CCHCR1* (a), *CDSN* (d) *DDAH2* (a)(b), *DDR1* (b), ENSG00000272221 (a)(b), ENSG00000272501 (b), ENSG00000288813 (b), *HCG27* (a), *HLA-B* (a)(c)(f), *HLA-C* (b)(c)(f), *HLA-S* (b), *MICA* (d), *MICB* (b), *MIR6891* (b), *PSORS1C1* (d), *PSORS1C3* (b), *TCF19* (b), *XXbac-BPG248L24.14* (g), CD4 + Tem HLA-DR+ (i), CD8 + Tc1/17 HLA-DR+ (i), mDC (j)

(a): eQTL in the 2000HIV study.

(b): eQTL in the GTEx.

(c): metQTL in Island or N/S-shore close to gene in the 2000HIV study.

(d): metQTL in OpenSea close to gene in the 2000HIV study.

(e): caQTL in or proximal to TTS in the 2000HIV study.

(f): caQTL in or proximal to TSS in the 2000HIV study.

(g): caQTL in intron/exon gene in the 2000HIV study.

(h): caQTL intergenic close to gene in the 2000HIV study.

(i): ccQTL in the 2000HIV study (absolute counts).

(j): ccQTL in the 2000HIV study (percentage of parent).

**Fig 4:**
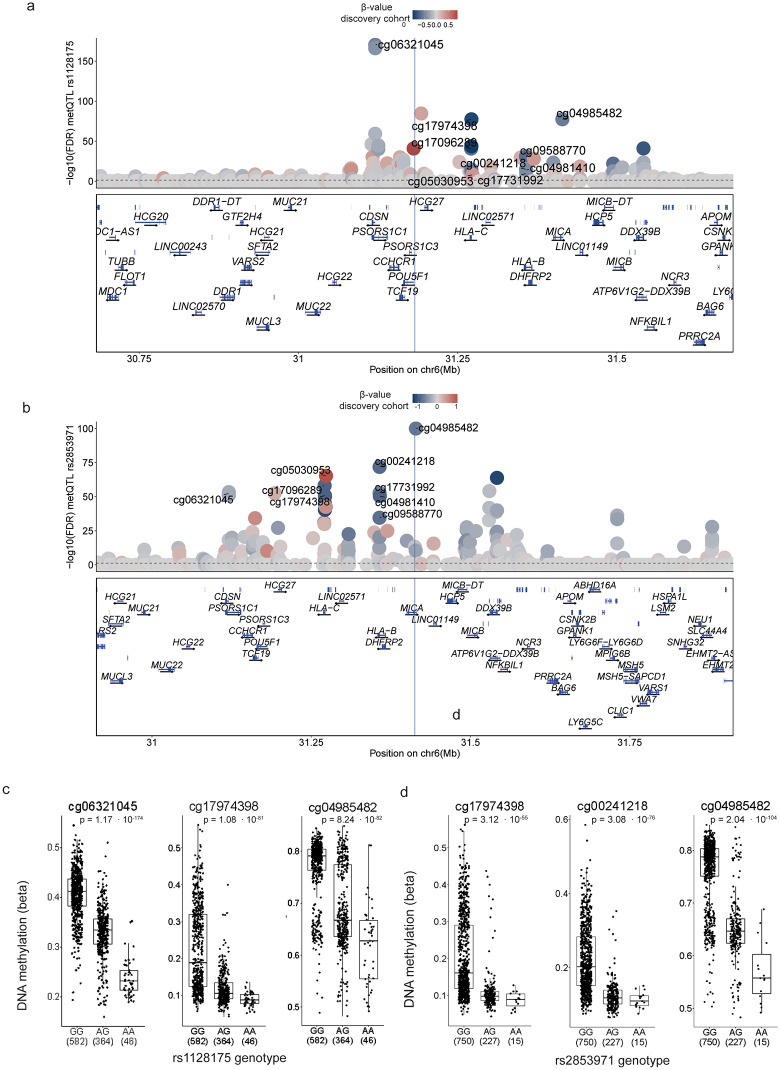
rs1128175 and rs2853971 influence DNA methylation in MHC locus. **(a + b)** Regional association plot of methylation quantitative trait loci (mQTLs) for rs1128175 **(a)** and rs2583971 **(b)**. The x-axis shows the chromosomal position, and the y-axis of the upper plot shows the –log_10_(P-value) for the mQTL. The color indicates the β value in the discovery cohort with regards to the A allele. The position of the SNP is indicated by the blue vertical line. **(c + d)** Genotype stratified boxplots for top mQTLs for rs1128175 **(c)** and rs2853971 **(d)**. The x-axis shows the genotype (count) and the y-axis represents the methylation beta value. The P-value in the discovery cohort is shown.

Third, to gain further insights into the mechanisms by which rs1128175 and rs2853971 influence gene expression, we assessed their effects on epigenetic modifications. For this, we tested whether these SNPs influence DNA methylation of CpGs in proximity to the SNPs (1 Mb window). Methylation QTL (mQTL) mapping showed that the rs1128175 and rs2853971 genotypes were significantly associated with the DNA methylation of 164 and 192 CpGs, respectively. The A allele of both SNPs was associated with decreased methylation near *MICA* and *CSDN,* in a CpG island near *HLA-C*, and in a CpG island and the south shore near *HLA-B* (Fig 4a-4d, S7 Table).

**Fig 5:**
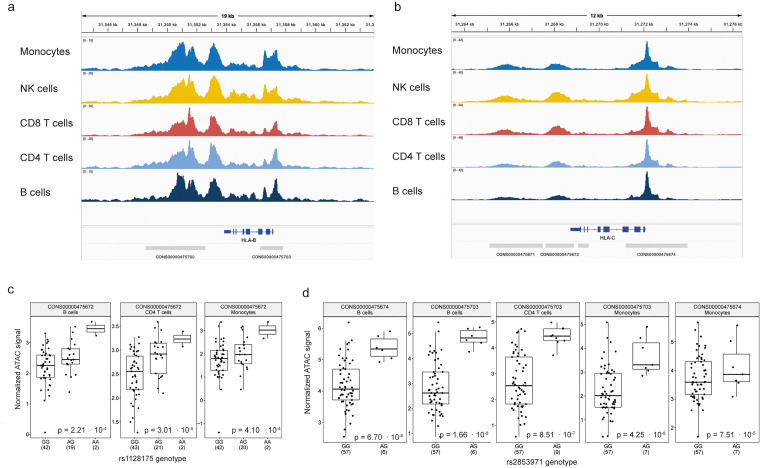
rs1128175 and rs2853971 influence chromatin accessibility in MHC locus. **(a + b)** Chromatin accessibility in the *HLA-B* **(a)** and *HLA-C* **(b)** locus. IGV plots showing chromatin accessibility profiles in monocytes, NK cells, CD8 T cells, CD4 T cells and B cells. Regions of interest and genes are annotated below the plot. **(c + d)** Genotype-stratified boxplots for significant caQTLs in B cells, CD4 T cells and monocytes for rs1128175 **(c)** and rs2853971 **(d)**.

Fourth, we assessed the effect of the two SNPs on chromatin accessibility in purified B cells, NK cells, CD8 T cells, CD4 T cells and monocytes, using ATAC-seq data from PLHIV of European ancestry (n_Bcell_ = 63, n_NKcell_ = 63, n_monocytes_ = 64, n_CD4Tcell_ = 66, n_CD8Tcell_ = 62). Chromatin accessibility QTL (caQTL) mapping showed that rs1128175 influences chromatin accessibility in B cells, CD4 T cells and monocytes, and the A allele was associated with increased chromatin accessibility downstream and at the transcription termination site of *HLA-C* (Fig 5b, 5c, S8 Table, S11 and S12 Figs). rs2853971-A, on the other hand, was associated with increased accessibility downstream of the promoter of *HLA-B* in all cell types. Additionally, it upregulates the accessibility at the transcription start site of *HLA-C* in B cells, NK cells, and monocytes (Table 1, Fig 5a, 5d, S8 Table, S11 and S12 Figs). Altogether, these findings provide additional evidence on the role of rs1128175 and rs2853971 in the HIV control phenotype and response to CMV, with their effects mediated by changes in DNA methylation and chromatin accessibility at specific sites in the MHC locus, ultimately regulating the expression of *HLA-B* and *HLA-C.*

Finally, we tested whether the SNPs influence immune cell composition in whole blood from PLHIV. We found that rs2853971-A was suggestively associated (P_discovery_ < 0.05 & P_validation_ < 0.05) with decreased absolute counts of CD8 Tc1/17 HLA-DR+ cells and CD4 Tem HLA-DR+ cells, and increased percentages of mDCs (Tables 1, S9). rs1128175-A was suggestively associated with increased percentages of CD8 Tc17 and PD-L1 + neutrophils (Tables 1, S9). Of these cell types, only CD8 Tc17 and PD-L1 + neutrophils showed a trend of being lower in HICs versus non-HICs, although not statistically significant, in consistency with the genetic effects (S9 Table). This suggests that rs1128175 and rs2853971 further influence the response to HIV and CMV by modulating the immune cell composition in PLHIV.

## Discussion

Earlier GWAS studies reported the impact of the MHC locus on the immunological control of HIV infection [13,14,35,36]. However, apart from the documented HIV specific effect [37,38], these HIV control associated genetic variants may also have functional immunological effects towards co-pathogens, such as CMV. In this study, we explored the impact of genetic variants associated with HIV control on cytokine production after PBMC stimulation with a CMV pp65 peptide pool and other pathogen-derived ligands. We found enrichment of genetic variants associated with HIV control in those regulating the cytokine response upon 24 hour PBMC stimulation with a CMV pp65 peptide pool, suggesting that responses to CMV and HIV control may have a common denominator. CMV is a very common co-pathogen in PLHIV, which persists in a latent state after the acute infection, from which it can reactivate as documented in both untreated and cART-exposed PLHIV [8,39–42]. Such reactivation may lead to presentation of CMV antigens, such as those derived from CMV pp65, that evoke immune memory responses.

Our analysis showed that the top enriched SNPs regulating the production of IL-1β and TNF-α upon stimulation with a CMV pp65 peptide pool were rs1128175 and rs2853971, respectively. The A alleles of both SNPs were linked to lower odds for HIV control (OR < 1) as well as increased cytokine production. Rs2853971-A showed a pleiotropic effect, upregulating TNF-α, IL-1β and MIP-1α production upon stimulation with a CMV pp65 peptide pool, suggesting that this variant influences a broader pro-inflammatory response to CMV pp65. The effect of these two variants was independent from the humoral response to CMV, as no interaction effect was found between the genetic associations with HIV control or cytokine responses and anti-CMV IgG titres. Our results are in line with previous studies that suggested that subclinical CMV reactivation and the associated inflammatory responses are associated with increased HIV persistence and reservoir size [8,12]. Together, these results link genetic variants associated with lower odds for HIV control to increased inflammatory responses to CMV pp65 with proposed negative impact on HIV control mechanisms.

We identified CD8 T cells and monocytes as the main source of TNF-α in PBMCs stimulated with a CMV pp65 peptide pool using intracellular cytokine staining, with increased production in CMV+ PLHIV. In contrast, the intracellular cytokine staining showed minimal IL-1β responses, although IL-1β is most likely produced by monocytes and DCs in the PBMC fraction [43]. This may be due to the shorter 4-hour stimulation in the intracellular cytokine staining experiments in contrast to the 24-hour PBMC stimulation studies with ELISA assay for detection of protein levels in supernatants. We must note here that, as it is unlikely that monocytes directly recognize CMV-derived peptide antigens, their activation likely occurs via memory CD8 T cells that recognize CMV pp65 peptides presented in the context of MHC class I. Indeed, it has been shown that antigen-specific CD8 T cells can induce IL-1β production in antigen-presenting cells through a perforin-mediated feedback mechanism *in vitro,* although the relevance of this mechanisms *in vivo* remains to be determined [44]. Additionally, the pp65-specific T cells produce IFN-γ, which can then prime monocytes and macrophages for the production of TNF-α and IL-1β [45]. These CMV pp65-specific CD8 T cells are known to be abundant in CMV+ PLHIV, with up to 20 percent of the CD8 T cells producing TNF-α and IFN-γ upon CMV pp65 stimulation [10]. Together, our data suggest that the pro-inflammatory response to CMV pp65 is driven by monocytes and CD8 T cells, with the CD8 T cell response potentially driving the monocyte response after recognition of the peptide antigen in the context of MHC class I.

The top enriched SNPs rs1128175 and rs2853971 are located within the MHC region, and are intronic variants in the *MICA* and *PSORS1C3* genes, respectively. The product of *MICA* gene is known to bind NKG2D, resulting in the activation of NK cells or co-stimulation of CD8 T cells while *PSORS1C3* is a previously described lncRNA associated with psoriasis susceptibility [46]. The rs1128175 variant is in moderate LD (r^2^ > 0.4) with two classical I MHC alleles, HLA-B*07:02 and HLA-C*07:02, and a proxy of rs2853971 is in strong LD (r^2^ > 0.8) with these classical I MHC alleles. This suggests that the effect of the SNPs on cytokine production and HIV control might be mediated by antigen presentation through MHC class I molecules. The association of HLA-B*07:02 and HLA-C*07:02 with poorer HIV control has previously been hypothesized to be mediated by poor antigen presentation capabilities of HIV antigens to CD8 T cells [13,14]. Differences in antigen presentation capabilities are influenced by specific amino acid residues within the HLA peptide groove, which are important for the binding and conformation of HIV epitopes. For example, a recent study using an engineered mutant HLA-B*57:01, where leucine at position 156 was substituted by the arginine (L156R), a residue known to be present in the HLA-B*07:02 risk allele, revealed the presence of HIV-specific CD8 T cells with significantly reduced recognition and elimination capacity for two different Gag epitopes, p24_108–117_ and p24_30–40_, but not for Nef_116–124_ [37]. Thus, HLA-B*07:02 and HLA-C*07:02 seem to have poor HIV Gag-specific antigen presenting capabilities.

In addition, our results suggest that the rs1128175-A/rs2853971-A/HLA-B*07:02/HLA-C*07:02 haplotype is associated with a higher inflammatory response to CMV pp65. This observation is further strengthened by the fact that HLA-B*07:02 and HLA-C*07:02 have been associated with increased IL-1β and TNF-α production upon CMV pp65 peptide pool stimulation in our 2000HIV cohort [31]. HLA-B*07:02 is known to present two immunodominant peptides derived from CMV pp65, known as a late CMV antigen. Our intracellular cytokine staining experiments showed that the rs1128175-A/rs2853971-A/HLA-B*07:02/HLA-C*07:02 haplotype is associated with increased IFN-γ production in CD8 T cells of PLHIV upon stimulation with a CMV pp65 peptide pool. We did not detect differences upon stimulation with an EBV BRLF1 peptide pool, indicating that the effect of the haplotype on IFN-γ production was specific to CMV pp65. HLA-B*07:02 has previously been associated with increased IFN-γ production upon CMV pp65 stimulation in HIV-negative individuals [32,47,48]. This suggests that the association of HLA-B*07:02 with the pro-inflammatory response to CMV pp65 is an intrinsic property of the HLA type, independent from the HIV control status. We therefore propose a model in which HLA-B*07:02 may shape pro-inflammatory responses to CMV prior to the establishment of HIV control status, rather than a model in which poor HIV control leads to increased pro-inflammatory responses to CMV. Given that HLA-C*07:02 is known to present an immunodominant epitope from CMV early antigen IE-1 [34,49], it does not exclude the possibility that its presence in the haplotype can also contribute to increased inflammatory responses to CMV.

Next, we assessed the effects of the top two enriched SNPs on gene expression, DNA methylation, chromatin accessibility and circulating immune cell composition to explore how these SNPs may co-regulate CMV-specific pro-inflammatory responses and HIV control. We observed that rs1128175-A and rs2853971-A were associated with the upregulation of classical MHC molecules *HLA-B* and *HLA-C,* likely through their effects on epigenetic modifications. Specifically, these alleles associated with increased chromatin accessibility at regions near or within the transcription start and termination sites of *HLA-B* and *HLA-C* in monocytes and CD4 T cells. During CMV co-infection in PLHIV, these are among the main cell types infected by HIV and CMV in the blood, respectively [50,51]. Additionally, we observed that these SNPs were associated with reduced DNA methylation upstream of the *HLA-B* and *HLA-C* gene. The top SNPs are thus associated with a more open chromatin state in the *HLA-B* and *HLA-C* locus, which might facilitate increased transcription. In contrast to our findings, previous studies showed that SNPs associated with a better HIV viral control led to increased *HLA-C* expression [52,53]. However, these studies did not consider the complex regulatory mechanisms of *HLA-C* expression in different cell types.

The modulation of HLA expression in CD4 T cells and monocytes and the ability to present antigens in infected cells is crucial in shaping the phenotype of cytotoxic NK and CD8 T cells [54,55]. Increased HLA expression on cell types infected by HIV and CMV allows for increased interaction with HIV and CMV-specific CD8 T cells. This increased interaction with T cell receptors of CMV-specific CD8 T cells, in combination with the favorable antigen-presenting properties of HLA-B*07:02 and HLA-C*07:02 for CMV, might shape a pronounced inflammatory CMV-specific CD8 T cell response. The HIV-specific CD8 T cell response, however, might not benefit from this increased HLA expression, given the poor antigen presentation capabilities of this HLA type for HIV antigens [38]. Additionally, increased HLA expression might lead to enhanced interactions with Killer Immunoglobulin Receptors (KIRs), in particular binding of HLA-C to KIR2DL2/3, which are expressed on NK cells and CD8 T cells. These enhanced interactions could lead to inhibition of NK and CD8 T cells, preventing killing of HIV-infected cells [56]. Thus, the long-term effect of epigenetic modifications on antigen presenting molecules, combined with antigen-presenting properties of HLA-B*07:02 and HLA-C*07:02, which are unfavorable for HIV Gag-epitopes, but favor pro-inflammatory responses to CMV pp65 epitopes, may be disadvantageous for HIV control.

Furthermore, rs1128175 and rs2853971 shape the CD8 T cell phenotype as they were found to influence the absolute counts and percentages of CD8 T cells. CD8 T cells are, in general, essential for killing responses and the establishment of HIV control, and HIV-specific CD8 T cells from HIV controllers were shown to have improved proliferative and effector capacities [22–25]. Additionally, rhesus macaques vaccinated with a CMV-vectored HIV vaccine were protected against progressive SIV infection, inducing strong effector memory CD8 T cell responses, in contrast to those vaccinated with an adeno-viral vectored vaccine, which elicited SIV-specific CD8 T cells with a central memory phenotype [57,58]. In particular, rs1128175-A is suggestively associated with increased percentages of CD8 Tc17 cells, and we found a trend towards lower percentages of these cell types in HICs compared to non-HICs. These cells are characterised by the production of IL-17A, which has a pro-inflammatory effect on monocytes, and often co-express pro-inflammatory cytokines, such as IFN-γ and TNF-α [59]. This general skewing of the CD8 T cell repertoire towards the Tc17 phenotype by rs1128175 might also affect CMV-specific T cells, favouring the pro-inflammatory response to CMV pp65. Overall, rs1128175 might steer the CD8 T cell repertoire towards a phenotype that may favour CMV-driven inflammation while it is not protective against HIV infection.

In conclusion, our results show that SNPs associated with reduced odds for HIV control are linked to an increased pro-inflammatory response to CMV pp65. These SNPs might modulate this inflammatory response through effects on antigen presentation, epigenetic modifications, gene expression, and the CD8 T cell phenotype. An increased pro-inflammatory response to CMV pp65 may favor HIV replication and reactivation, predisposing individuals to the non-controller phenotype. Future studies should focus on elucidating the relationship between inflammatory responses to CMV and HIV control, which would help build strategies targeting CMV-driven inflammation in the context of HIV functional cure studies.

## Materials and methods

### Ethics statement

This study was approved by the medical-ethical research committee Nijmegen (NL68056.091.81). All participants gave written informed consent prior to participation in the study. All experiments with human samples were conducted in line with the regulations of the Declaration of Helsinki.

### Cohorts

1,895 PLHIV were included in four medical centers in the Netherlands as part of the 2000HIV study (clinicaltrials.gov NCT03994835) between November 2019 and October 2021 and divided into a discovery (n = 1,559) and independent validation cohort (n = 336). The 2000HIV study is part of the Human Functional Genomics Project [60]. All participants received cART, with the exception of a subgroup of HIV controllers. Inclusion criteria, visit procedure and sample collection and storage were conducted as previously described [61].

Within the 2000HIV study, HICs were included, characterized by a current or historical capacity to spontaneously control HIV-1. Within the literature, many definitions are used for this group. We included two types of HICs: the elite controllers (ECs) and the viremic controllers (VCs). ECs were defined as individuals that had HIV-1 RNA load < 75 copies/ml and stable (i.e., > 75% of measurements) CD4 T cell counts of at least 500 cells/mm^3^ for at least 12 months in the absence of cART. VCs were defined as individuals with an HIV-1 RNA load of < 10,000 copies/ml and stable CD4 counts of at least 500 cells/mm^3^. While this viral load threshold is on the higher end of those reported, it is more commonly used in literature [5]. To ensure the inclusion of true controllers, we have extended the duration for viral control to a minimum of five years. Individuals who met the criteria for HIV control at the time of inclusion were considered persistent controllers, while those who met the criteria previously but lost control before inclusion were considered transient controllers. The HICs in the validation cohort were mostly transient controllers and their number was small, restricting the use of this group to validate findings in HICs compared to non-HICs.

### GWAS on HIV control

The Genome-wide association study (GWAS) was performed in 67 HIC and 1,179 non-HIC of European ancestry. DNA was isolated from whole blood and participants from all ancestries were genotyped using the Illumina Infinium Global Screening Array. Pre-imputation quality control (QC) on raw variants and samples was performed using PLINK v1.90b [62]. Genetic variants with a missingness of more than 5% and those deviating from Hardy-Weinberg equilibrium (HWE) with a P-value of < 10^-6^ were excluded from analysis. The HWE exact test was performed stratified by ancestry. Samples with a call rate < 97.5% and those with heterozygosity rates that deviated more than three standard deviations (SD) from the mean rate per ancestry were excluded. Genetic variants that passed QC were lifted from GRCh37 to GRCh38 using the UCSC liftOver tool [63]. The variants were aligned to the TOPMed reference panel (Freeze 5) on GRCh38 using the McCarthy group tools (https://www.well.ox.ac.uk/~wrayner/tools/). In total, 582,404 variants and 1,864 individuals passed QC and were kept for imputation. Genotypes of variants not included in the array were inferred by imputation against the TOPMed panel (v2 on GRCh38), using the TOPMed imputation server. The imputed variants were then filtered using BCFtools, removing variants with a low imputation quality (R2 < 0.3 or ER2 < 0.7) and those with minor allele frequency (MAF) < 1%. This resulted in a dataset of 10,810,841 variants from 1,864 individuals. Since population stratification is an important confounder in genetic studies, additional post-imputation quality control was performed for individuals of European ancestry only. PLINK 1.90b was used for this post-imputation QC. First, variants with a MAF < 0.05 and those deviating from HWE (P < 10^-6^) were excluded. Second, individuals with outlying heterozygosity (> 3 standard deviations (SD) from mean heterozygosity rate), one individual with discordant sex information (X chromosomal inbreeding coefficient > 0.2 for females or < 0.8 for males), and those with evidence for relatedness (identity-by-descent > 0.2) were removed. Third, ancestral outliers were identified using principal component analysis (PCA), and those deviating more than three SDs from the 1000genomes European reference population on principal component 1 or 2 were excluded. In total, 5,791,803 SNPs with minor allele frequency (MAF) > 5% were tested for association with HIV control using a logistic regression model with age, sex and the first five genetic principal components (PCs) as covariates with PLINK v1.90b [62]. The genetic PCs were included in the model to account for population stratification within the cohort.

### *Ex vivo* cytokine production in PLHIV

To assess *ex vivo* cytokine production, PBMCs from the participants were isolated by density gradient separation and seeded in cell culture plates at a density of 500.000 cells/well. The cells were stimulated with either RPMI as control or peptide pools of viral proteins HIV-ENV and CMV pp65, Toll-like receptor (TLR)-agonists imiquimod (IMQ), lipopolysaccharide (LPS) and polyiosinic-polycytidylic acid (Poly:IC), bacterial stimulus *Streptococcus pneumoniae*, and the alarmin interleukin (IL)-1α for 24 hours. Additional stimulations with fungal pathogen *C. albicans* hyphae and conidia, bacteria *Eschericia coli*, *Mycobacterium tuberculosis*, *Staphylococcus aureus* and *S.pneumoniae* and mitogen phytohaemagglutinin (PHA) were performed for 7 days. Cell culture supernatants were stored at -20° C and concentrations of relevant cytokines were measured by ELISA. To exclude false RPMI positive measurements, individuals with a cytokine concentration of at least 2 times the lower limit of detection in the negative control (RPMI only) for at least two out of IL-1β, IL-6 and TNF-α were removed from the analysis. Principal component analysis (PCA) was used to detect outlier samples, and samples that deviated over 4 standard deviations from the mean of all samples on either PC1 or PC2 were removed.

### Cytokine-QTL summary statistics

We used the summary statistics of cytokine-QTLs (cQTLs) identified in PLHIV as part of the 2000HIV study [30]. In short, imputed genome-wide genetic data and *ex vivo* cytokine responses from the 2000HIV study were used for cQTL mapping. Inverse-rank transformed cytokine responses were mapped to the genotyped data using a linear model correcting for age, sex, BMI, seasonality, inclusion after the COVID-19 pandemic, COVID-19 vaccination and recruitment centre. QTL mapping was performed in the 2000HIV discovery and validation cohort separately. Genotype-stratified boxplots include all individuals of European ancestry that passed genetic QC in the discovery cohort.

### Enrichment analysis

To test for enrichment of HIV-control associated SNPs in cQTLs, cQTLs for all cytokine-stimuli pairs were extracted using the summary statistics of the 2000HIV discovery cohort. Due to limited statistical power, we extracted cQTLs at three different suggestive P-value thresholds (P < 1 ∙ 10^-5^, P < 1 ∙ 10^-4^, and P < 1 ∙ 10^-3^) to ensure that this enrichment was not dependent on the threshold that was chosen. These cQTLs were overlaid with the GWAS summary statistics on HIV control. The lambda inflation was calculated based on the GWAS P-values for all cQTLs that passed the respective P-value threshold, regardless of whether the GWAS P-value was significant. Lambda values were calculated as the median chi-square statistic of the association with HIV control divided by the median chi-square statistic of the uniform distribution. Cytokine-stimuli pairs with a lambda inflation of > 1.05 were considered inflated while those with a lambda value below < 0.99 were considered deflated. For visualization, expected GWAS P-values were calculated using the *ppoints* function based on the number of cQTLs that passed the significance threshold for the respective cytokine-stimulus pair. Because of the low number of cQTLs for some cytokine-stimuli pairs, cases showed an inflated lambda value, suggesting enrichment, whereas the QQ plot suggested deflation. For enrichment of HIV-control associated SNPs in cQTLs, we thus considered only cytokine-stimuli pairs with consistent inflation based on the lambda value and visual inspection of the QQ plots.

To ensure robustness of the enrichment of HIV-control associated SNPs in cQTLs for IL-1β and TNF-α upon stimulation with a CMV pp65 peptide pool, we performed 500 permutations. In each permutation, for each cQTL SNP of interest that passed the significance threshold (P < 1 ⋅ 10^-5^), we selected independent SNPs located within a 500 kb or 1 Mb window, with a similar MAF (+- 0.05) and not in LD with the original cQTL (r^2^ < 0.1). Using these matched SNPs, we recalculated the lambda value for each permutation and calculated the 95% confidence interval across all permutations. The enrichment was considered robust if the actual lambda value fell outside of the 95% confidence interval.

### CMV serology

In the 2000HIV cohort, CMV serostatus and plasma anti-CMV IgG titres were determined using the CMV IgG enzyme immunoassay kit (GenWay, catalog number GWB-892399) on plasma from all individuals as per the manufacturer’s instructions.

### Interaction model anti-CMV IgG titres

The effect of anti-CMV IgG titres on the genetic associations with HIV control and IL-1β and TNF-α cytokine production upon stimulation with a CMV pp65 peptide pool was assessed using by adding an interaction term for anti-CMV IgG titres to the models described above. We used the following models for HIV control and *ex vivo* cytokine production, respectively:


$$\begin{array}{c} HIC\;status\;\sim \;age+sex+genetic\;PC\text{1}-\text{5}+anti-CMV\;IgG\;titers+SNP+SNP\times \;anti-CMV\;IgG\;titres \end{array}$$

$$\begin{array}{cc} & Cytokine\;production\;\sim \;age+sex+bmi+seasonality+lockdown+covid_{vaccination}+covid_{infection}+inclusion\;center\\ & +anti-CMV\;IgG\;titers+SNP+SNP\times \;anti-CMV\;IgG\;titres \end{array}$$

CMV seronegative individuals and those without anti-CMV IgG titres available were excluded from analysis. The GWAS on HIV control was performed using 59 HICs versus 1,157 non-HICs in plink v1.90b [62]. cQTL mapping was conducted using MatrixEQTL including 942 and 719 individuals with measurements of IL-1β and TNF-α production, respectively [64].

### MHC imputation

After standard QC procedures, SNPs in the MHC region, amino acids and HLA alleles located on chromosome 6 were imputed on the Michigan imputation server based on the four-digit multi-ethnic HLA v2 reference panel. Linkage disequilibrium between SNPs and classical HLA alleles was calculated in 1,271 individuals of European ancestry using PLINK v1.90b and the 2000HIV as a reference panel. Only classical MHC alleles with a moderate LD (r^2^ > 0.4) with the SNPs, rs1128175 and rs2853971, were reported.

### Intracellular cytokine staining

Frozen PBMCs of 10 PLHIV (CMV 5 seronegative and 5 seropositive) were seeded at 1 ∙ 10^6^ cells per condition and cultured 18 hours at 37°C in RPMI 1640 (Dutch Modification; Gibco) supplemented with 10% heat-inactivated human pooled serum. Cells were stimulated with a CMV pp65 peptide pool (1 μg/mL; JPT Peptide Solutions) and with LPS (10 ng/mL; Sigma) or PMA (50 ng/mL; Sigma)/Ionomycin (1 μg/mL; Sigma) as positive controls. After 1 h of incubation at 37°C, 60 µg/mL Brefeldin A (Sigma) was added to the culture and incubated for another 3 hours. Afterwards, cells were washed and stained with fixable viability dye (ViaKrome 808; Beckman Coulter) for 20 min at room temperature (RT). After washing cells were stained with antibodies against surface markers: KrO-CD45 (Beckman Coulter; clone J33), APC-CD8 (Biolegend; clone RPA-T8), AF700-CD14 (Biolegend; clone HCD14), PCy5-CD19 (Biolegend; clone HIB19), BV605-CD3 (BD; clone HIT3alpha), BUV395-CD4 (eBioscience; clone SK-3), BV-737-CD56 (BD, NCAM16.2) for 20 min at RT. Cells were washed and fixed with Foxp3 fixation/permeabilization buffer (eBioscience) for 30 min at 4°C. After washing with permeabilization buffer (eBioscience) cells were stained for intracellular cytokines with PE-Cy7-IFNg (Biolegend; clone 4S.B3 – Panel 1), PE-Cy7-TNF (Biolegend; clone Mab11 – Panel 2) and FITC-IL-1b (Biolegend; H1b-98 – Panel 2) for 30 min at 4°C. After washing with permeabilization buffer, cells were resuspended in PBS with 1% bovine serum albumin (BSA; Sigma) and acquired on a Cytoflex LX (Beckman Coulter) the same day.

In addition, to assess the effects of the rs1128175-A/rs2853971-A/HLA-B*07:02/HLA-C*07:02 haplotype on intracellular IFN-γ expression, we performed the assays described above using PBMCs (3 × 10⁵ cells per condition) from 11 PLHIV, of whom 5 non-HICs carried the rs1128175-A/rs2853971-A/HLA-B*07:02/HLA-C*07:02 haplotype and 6 HICs did not. Cells were stimulated with a CMV pp65 peptide pool and an EBV BRLF1 peptide pool (1 μg/mL; JPT Peptide Solutions). From the same donors, CD8 T cells were isolated by MACS microbeads (positive selection; Miltenyi Biotec), yielding CD8 T cell-enriched and CD8 T cell–depleted fractions. Both fractions were seeded at 2 ∙ 10⁵ cells per condition and stimulated with a CMV pp65 peptide pool (1 μg/mL; JPT Peptide Solutions), followed by intracellular IFN-γ assessment by flow cytometry as described above. Flow cytometry data was analysed using Kaluza software (version 2.2.0). Compensation matrices were constructed and FMO controls were used to determine positive and negative populations for IFN-γ,TNF-α and IL-1β.Gating strategy and representative plots are shown in S13 and S14 Figs.

### DNA methylation profiling and QC

DNA was isolated from whole blood of 1,870 participants using the ChemagicStart system. DNA methylation was assessed using the Illumina Infinium MethylationEPIC v1 BeadChip array. DNA methylation quality control steps were performed for the entire cohort, following the steps as described earlier [65]. DNA methylation M-values and β-values were calculated from the original IDAT files using the minfi package [66]. Samples with sex mismatches and those with a call rate < 0.99 were removed from analysis. Probes with missing values in over 10% of the samples and those on the sex chromosomes were removed. Additionally, probes containing SNPs with a MAF > 5% in the European population at the target CpG site and those mapping to multiple loci were removed. Subsequently, stratified quantile normalisation was applied to the methylation data. The methylation values were used to estimate the proportions of six immune cell types using the ‘estimateCellCounts2’ function from the FlowSortedBlood_EPIC package [67]. After quality control, 2,091 and 2,557 *cis*-CpGs were retained in a 1 Mb window (+- 500 kb) from rs1128175 and rs2853971, respectively.

### Profiling and QC of bulk gene expression

RNA was extracted from PBMCs using TRIzol-chloroform extraction, after which libraries were generated using the Illumina Stranded mRNA Prep kit. Short read sequencing (paired end, 15 million reads per sample) was performed using the Illumina NovaSeq 6000 machine. Reads were mapped to the human reference genome (GRCh38, gencode version 33) and genes were annotated with their respective Ensembl ID (version 33). During quality control, samples with less than 5 million reads mapped, those with discrepancy in biological sex and Chr-Y expression were removed. This resulted in a total of 1,853 samples and 58,347 transcripts available for analysis in the entire cohort.

### Chromatin accessibility profiling and QC

CD14+ monocytes, CD19+ B cells, CD56+ NK cells, CD4+ T cells, CD8+ T cells were enriched from PBMCs using MACS kit (Microbeads human, positive selection, Miltenyi Biotec), according to the manufacturer’s instructions. The enriched cells were further processed for ATAC-sequencing. Briefly, 50.000 cells were washed once in 50 μl cold PBS and lysed in a transposase reaction mix containing 10.25 μl nuclease free water, 12.50 μl 2 × Tagment DNA Buffer (Illumina), 2 μl Tagment DNA Enzyme (Illumina), and 0.25 μl 1% digitonin (Promega) for 30 minutes at 37 °C. Upon DNA purification with the MinElute kit (Qiagen), 1 μl of eluted DNA was used in a qPCR reaction in order to estimate the optimum number of amplification cycles. Next, the remaining 10 μl of each library was amplified for the number of cycles corresponding to the Cq value (the cycle number at which fluorescence has increased above background levels) from the qPCR. Library amplification was followed by SPRI (Beckman Coulter) size selection to exclude fragments exceeding 1000 bp in length. DNA concentration was measured with a Qubit fluorometer (Life Technologies). Library amplification was performed using custom Nextera primers. Libraries were sequenced by the Biomedical Sequencing Facility at CeMM using the Illumina NovaSeq 6000 S2 platform and the 100-bp paired-end configuration.

*Processing.* Sequencing adapters were removed using the software fastp (v0.23.2) [68]. Bowtie2 (v2.4.5) [69] was used for the alignment of the short reads (representing locations of transposition events) to the GRCh38 (hg38) assembly of the human genome using the “--very-sensitive” parameter. PCR duplicates were marked using samblaster (v0.1.24) [70]. Aligned BAM files were then sorted, filtered using ENCODE blacklisted regions (v3) [71], and indexed using samtools (v1.12) [72]. To detect open chromatin regions, peak calling was performed using MACS2 (v2.2.7.1) [73] using the “--nomodel”, “--keep-dup auto” and “--extsize 147” options on each sample. Quality control metrics were aggregated and reported using MultiQC (v1.9) [74], and one sample needed to be removed because of low read counts.

*Quantification.* A consensus region set, comprising of 1,220,778 genomic regions, was generated, by merging the identified peak summits, extended by 250 bp on both sides using the slop function from bedtools (v2.27.1) [75] and pybedtools (v0.9.0) [76], across all samples while again discarding peaks overlapping blacklisted features as defined by the ENCODE project [71]. Consensus regions were annotated using annotatePeaks function from HOMER (v4.11) [77]. Leading to a common consensus set of regions that is can be filtered for each cell-type and study.

Additionally, we annotated all consensus regions using UROPA [78] and genomic features from the [GENCODE v38] basic gene annotation. Three queries were used to define regions in/near the TSS in the UROPA configuration file. Specifically, these were:

1. “TSS” = regions with the peak center within 100 bp of the start of a gene
2. “TSS_proximal” = regions with the peak center 1,000 bp upstream to 500 bp downstream of the start of a gene
3. “TSS_overlap” = regions with the peak center within 10,000 bp of the start of a gene and overlapping the start of the gene
4. “TSS_FIP” = regions which entirely contain a gene with the peak center within 10,000 bp of the start of a gene

The consensus region set was used to quantify the chromatin accessibility in each sample by summing the number of reads overlapping each consensus region. The consensus region set, and sample-wise quantification of accessibility was performed using bedtools (v2.27.1) [75] and pybedtools (v0.9.0) [76].

*Downstream Analysis.* For downstream analyses, we filtered the 1,220,778 consensus regions to 271,379 regions using the EdgeR function “filterByExpr”, using the following settings:

- *min.count* = 50
- *min.prop* = 0.9,
- *min.total.count* = numberOfSamples*10
- *large.n* = round(max(table(*group*))*.5, digits = 0)

where “*numberOfSamples”* is the number of samples in the data and “max(table(*group*))” computes the number non-HICs in the data.

The processing and analysis described here was performed using a publicly available Snakemake [v7.2] workflow (https://github.com/epigen/atacseq_pipeline).

### Phenotyping of circulating immune cell subsets

The phenotype of circulating immune cells was assessed through flow cytometry, using three panels focusing on the characterization of innate, T and B cells in whole blood. The immunophenotyping approach from the 2000HIV study, including sample processing, acquisition, and gating strategies was performed as described previously [79]. In total, 355 manually annotated immune cell populations were included in the analysis.

### Expression-QTL summary statistics

To assess the effect of rs1128175 and rs2853971 on gene expression, we used summary statistics of eQTLs from the 2000HIV study, which capture associations between SNP genotypes and gene expression levels [30]. eQTL mapping was performed in 1,048 and 260 PLHIV in the discovery and validation cohorts, respectively. Statistical modelling was identical to the cQTL mapping as described above.

### Statistical analysis and visualization

Statistical analysis and visualization were performed in R version 4.4.3. For intracellular cytokine staining experiments, differences between percentages of cell types within the CMV serostatus group before and after stimulation were assessed using a Wilcoxon signed-rank test. A Wilcoxon rank-sum test was used to compare percentages between CMV serostatus groups.

RNA sequencing differential expression analysis (DEA) was performed to test for differences in RNA expression patterns in PBMCs from 96 HICs and 1,363 non-HICs in the 2000HIV discovery cohort. The DESeq2 workflow [80] was used for transcriptomics analysis, applying negative binomial generalized linear models adjusted for sex, center of collection, age, seasonality scores, plate effects, and the first five genetic principal components to account for ethnicity. Log2 fold change (Log2FC) shrinkage was performed using the “apeglm” method. Genes with a nominal P-value < 0.05 were considered suggestively associated with the HIC phenotype.

### Quantitative trait locus mapping

Associations between rs1128175 and rs2853971 genotypes and molecular traits were tested as follows:

DNA methylation M-values in a 1 Mb window of the SNPs were modeled as a function of genetic dosages using linear regression adjusting for relevant covariates (see table below), using MatrixEQTL [64]. Methylation QTL (mQTL) mapping was performed separately in the discovery and validation cohorts and mQTLs with a Benjamini-Hochberg corrected FDR < 0.05 in the discovery cohort and a nominal P-value < 0.05 in the validation cohort were considered statistically significant.

For chromatin-accessibility QTLs (caQTL), the discovery and validation cohorts were combined given the limited number of samples. Normalized library sizes were calculated using the edgeR::CalcNormFactors function with the trimmed mean of M values (TMM) method [81]. QTL mapping was performed using the LIMMA package [82], applying the voom function to calculate mean-variance relationship, lmFit for linear modelling and eBayes moderation for the calculation of t-statistics. Only features within a 1 Mb window of the SNPs, rs1128175 and rs2853971, were mapped, and those with a Benjamini-Hochberg corrected FDR < 0.05 were considered statistically significant.

Cell count QTL (ccQTL) mapping was performed similar to mQTL mapping on inverse-rank transformed absolute counts and percentages of parents. ccQTL mapping was performed separately in the discovery and validation cohorts, and ccQTLs with a nominal P-value < 0.05 in both discovery and validation cohort were considered as suggestive associations.

**Table:** 

*Data layer*	*Method*	*Number of individuals*	*Covariates*
DNA methylation	MatrixEQTL	Discovery cohort: 1,072 Validation cohort: 266	Age, sex, sample plate, proportions of CD8 T cells, CD4 T cells, NK cells, B cells, Monocytes and Neutrophils
Chromatin accessibility – B cells	LIMMA	63	Age, sex, inclusion center, frip, seasonality, and the first 3 genetic PCs
Chromatin accessibility – NK cells		63	
Chromatin accessibility – CD4 T cells		66	
Chromatin accessibility – CD8 T cells		62	
Chromatin accessibility – monocytes		64	
Cell counts	MatrixEQTL	Discovery cohort: 725 Validation cohort: 178	Age, sex, seasonality, COVID-19 vaccination, and the first five genetic PCs

Abbreviations: PCs: Principal Components.

## Supporting information

S1 Fig Overview of assessed cytokine-stimuli pairs in the ex vivo cytokine-QTL mapping summary statistics used in this study. (PDF)

S2 Fig Enrichment of HIV control SNPs in suggestive (P < 1 · 10^-3^) cQTLs in the 2000HIV cohort. (a) Enrichment results for cQTLs for cytokine production measured after 24 hours of stimulation. (b) Enrichment results for cQTLs for cytokine production measured after 7 days of stimulation. Enrichment results are split per stimulus (horizontal) and cytokine (vertical), grey squares represent cytokine-stimuli pairs that were not measured. Shown are SNPs that pass the suggestive significance threshold in the cQTL mapping for the respective cytokine-stimulus pair. The x-axis shows the expected -log_10_(P-value) under a uniform distribution. The y-axis represents the actual -log_10_(P-value) in the GWAS for HIV control. Lambda values for genomic inflation are shown. Blue boxes indicate cytokine-stimuli pairs for which enrichment of HIV control associated SNPs was found in cQTLs at P < 1 ⋅ 10^-3^. Red boxes indicate cytokine-stimuli pairs for which consistent enrichment of HIV control associated SNPs in the cQTLs was found over 3 P-value thresholds.
(TIF)

S3 Fig Enrichment of HIV control SNPs in suggestive (P < 1 · 10^-4^) cQTLs in the 2000HIV cohort. (a) Enrichment results for cQTLs for cQTLs for cytokine production measured after 24 hours of stimulation. (b) Enrichment results for cytokine production measured after 7 days of stimulation. Enrichment results are split per stimulus (horizontal) and cytokine (vertical), grey squares represent cytokine-stimuli pairs that were not measured. Shown are SNPs that pass the suggestive significance threshold in the cQTL mapping for the respective cytokine-stimulus pair. The x-axis shows the expected -log_10_(P-value) under a uniform distribution. The y-axis represents the actual -log_10_(P-value) in the GWAS for HIV control. Lambda values for genomic inflation are shown. Blue boxes indicate cytokine-stimuli pairs for which enrichment of HIV control associated SNPs was found in cQTLs at P < 1 ⋅ 10^-4^. Red boxes indicate cytokine-stimuli pairs for which consistent enrichment of HIV control associated SNPs in the cQTLs was found over 3 P-value thresholds.
(TIF)

S4 Fig Enrichment of HIV control SNPs in suggestive (P < 1 · 10^-5^) cQTLs in the 2000HIV cohort. (a) Enrichment results for cQTLs for cytokine production measured after 24 hours of stimulation. (b) Enrichment results for cQTLs for cytokine production measured after 7 days of stimulation. Enrichment results are split per stimulus (horizontal) and cytokine (vertical), grey squares represent cytokine-stimuli pairs that were not measured. Shown are SNPs that pass the suggestive significance threshold in the cQTL mapping for the respective cytokine-stimulus pair. The x-axis shows the expected -log_10_(P-value) under a uniform distribution. The y-axis represents the actual -log_10_(P-value) in the GWAS for HIV control. Lambda values for genomic inflation are shown. Blue boxes indicate cytokine-stimuli pairs for which enrichment of HIV control associated SNPs was found in cQTLs at P < 1 ⋅ 10^-5^. Red boxes indicate cytokine-stimuli pairs for which consistent enrichment of HIV control associated SNPs in the cQTLs was found over 3 P-value thresholds.
(TIF)

S5 Fig Results of permutations to assess robustness of enrichment of HIV control SNPs in cQTLs for IL-1β (a,c) and TNF-α (b,d) responses to CMV pp65. For each of the 500 permutations, for each cQTL that passed the suggestive threshold (P < 1 • 10^-5^), a random SNP was selected within 500kb (a,b) or 1 Mb (c,d) distance, with a similar MAF (+- 0.05), not in LD with the original SNP (r^2^ < 0.1). Using these randomly selected SNPs, the lambda value was recalculated. Yellow histograms represent the distributions of these lambdas, and the blue shading represents the 95% confidence interval. The red dashed line represents the lambda value in the original enrichment analysis.
(TIF)

S6 Fig Pleiotropic effects of rs2853971 and rs1128175 on ex vivo cytokine production. The x-axis shows all cytokine-stimuli pairs for which suggestive associations (P_discovery_ < 0.05) were found and the y-axis shows the two top enriched SNPs. The size of the dots indicates the -log_10_(P-value) in the discovery cohort and the color indicates the beta value with respect to the HIV control risk allele, with red indicating that the HIV control risk allele is associated with increased cytokine production. * indicates an effect (P_discovery_ < 3.2 • 10^-4^) ** indicates an effect (P_discovery_ < 3.2 • 10^-4^) validated in the validation cohort (P_validation_ < 0.05).
(TIF)

S7 Fig Cellular composition and intracellular expression of TNF-α and IL-1β upon PBMC stimulation with LPS in CMV+ and CMV- PLHIV. (a) Schematic overview of the experimental design. PBMCs from 10 PLHIV (5 CMV+ and 5 CMV-) were stimulated with RPMI (negative control) or LPS (positive control) for 4 hours, of which 3 hours in the presence of brefeldin A. The cellular composition and intracellular TNF-α and IL-1β expression in CMV+ and CMV- individuals were assessed using flow cytometry. (b) Immune cell subsets B cells (CD19+), CD4 T cells (CD3 + CD4 + CD8-), CD8 T cells (CD3 + CD4-CD8+), monocytes (CD14+), NK cells (CD3-CD56+), and NKT cells (CD3 + CD56 + CD8-) as percentage of total CD45 + cells measured. (c + d) Percentage of TNF-α+ and IL-1β+ cells within each cell subset, respectively. Bar heights represent mean percentages, and color represents CMV serostatus; CMV- in blue and CMV+ in yellow. P-values < 0.10 from Wilcoxon rank-sum test (for comparisons between CMV serostatus groups) and Wilcoxon signed-rank test (for comparisons within CMV serostatus groups) are shown.
(TIF)

S8 Fig Cellular composition and intracellular expression IL-1β upon PBMC stimulation with a CMV pp65 peptide pool in CMV+ and CMV- PLHIV. (a) Schematic overview of the experimental design. PBMCs from 10 PLHIV (5 CMV+ and 5 CMV-) were stimulated with RPMI (negative control) or a CMV pp65 peptide pool for 4 hours, of which 3 hours in the presence of brefeldin A. The cellular composition and intracellular IL-1β expression in CMV+ and CMV- individuals were assessed using flow cytometry. (b) Immune cell subsets B cells (CD19+), CD4 T cells (CD3 + CD4 + CD8-), CD8 T cells (CD3 + CD4-CD8+), monocytes (CD14+), NK cells (CD3-CD56+), and NKT cells (CD3 + CD56 + CD8-) as percentage of total CD45 + cells measured. (c) Percentage IL-1β+ cells within each cell subset. Bar heights represent mean percentages, and color represents CMV serostatus; CMV- in blue and CMV+ in yellow. P-values < 0.10 from Wilcoxon rank-sum test (for comparisons between CMV serostatus groups) and Wilcoxon signed-rank test (for comparisons within CMV serostatus groups) are shown.
(TIF)

S9 Fig Cellular composition and intracellular expression of IFN-γ upon PBMC stimulation with PMA/ionomycin in CMV+ and CMV- PLHIV. (a) Schematic overview of experimental procedure. PBMCs from 10 PLHIV (5 CMV+ and 5 CMV-) were stimulated with RPMI (negative control) or PMA/ionomycin (positive control) for 4 hours, of which 3 hours in the presence of brefeldin A. The cellular composition and intracellular IFN-γ expression in CMV+ and CMV- individuals were assessed using flow cytometry. (b) Immune cell subsets B cells (CD19+), CD4 T cells (CD3 + CD4 + CD8-), CD8 T cells (CD3 + CD4-CD8+), monocytes (CD14+), NK cells (CD3-CD56+), and NKT cells (CD3 + CD56 + CD8-) as percentage of total CD45 + cells measured. (c) Percentage of IFN-γ+ cells within each cell subset. Bar heights represent mean percentages, and color represents CMV serostatus; CMV- in blue and CMV+ in yellow. P-values < 0.10 from Wilcoxon rank-sum test (for comparisons between CMV serostatus groups) and Wilcoxon signed-rank test (for comparisons within CMV serostatus groups) are shown.
(TIF)

S10 Fig Intracellular cytokine staining for IFN-γ upon stimulation with CMV pp65 and EBV BRLF1 peptide pools. (a) Schematic overview of the experiment. PBMCs from 5 non-HICs carrying the rs1128175-A/rs2853971-A/HLA-B*07:02/HLA-C*07:02 haplotype (Hap+) and 6 HICs not carrying the haplotype (Hap-) were stimulated with a CMV pp65 or EBV BRLF1 peptide pool for 4 hours, of which 3 in the presence of brefeldin A. IFN-γ production was measured intracellularly by flow cytometry in six major cell populations: B cells (CD19+), CD4 T cells (CD3 + CD4 + CD8-), CD8 T cells (CD3 + CD4-CD8+), monocytes (CD14+), NK cells (CD3-CD56+), and NKT cells (CD3 + CD56 + CD8-). (b) Percentage of IFN-γ+ cells within each population (y-axis) stratified by stimulus (x-axis). Colors represent the stimulus. (c) Percentage of IFN-γ positive CD4 and CD8 T cells (y-axis) upon stimulation with EBV BRLF1 or RPMI as a control. Colors represent the presence of the haplotype. Statistical testing was performed using a Wilcoxon signed-rank test for comparisons within haplotype groups and a Wilcoxon rank-sum test for comparison between haplotype groups. Only P-values < 0.10 are shown.
(TIF)

S11 Fig Chromatin accessibility profiles of regions influenced by rs1128175 (a) and rs2853971 (b). ATAC-seq counts are visualized for all individuals included in the ATAC-seq experiment per cell type. The main peaks for which caQTLs were found are labelled in the respective plot.
(TIF)

S12 Fig Boxplots of all significant caQTL for rs1128175 and rs2853971. The x-axis shows the genotypes (count) and the y-axis the normalized chromatin ATAC sequencing signal.
(TIF)

S13 Fig General gating strategy applied on PBMCs to determine the percentages of B cells (CD45 + CD3-CD19+), CD4 and CD8 T lymphocytes (CD45 + CD3 + CD4+) and (CD45 + CD3 + CD8+), monocytes (CD45 + CD14+), Natural killer (NK) (CD45 + CD3-CD56+) and NK-T (CD3 + CD45 + CD8-) producing IFN-γ upon CMV pp65 stimulation (Panel 1). (PDF)

S14 Fig General gating strategy applied on PBMCs to determine the percentages of B cells (CD45 + CD3-CD19+), CD4 and CD8 T lymphocytes (CD45 + CD3 + CD4+) and (CD45 + CD3 + CD8+), monocytes (CD45 + CD14+), Natural killer (NK) (CD45 + CD3-CD56+) and NK-T (CD3 + CD45 + CD8-) producing TNF-ɑ and IL-1β upon CMV pp65 stimulation (Panel 2). (PDF)

S1 Table cQTL summary statistics for cQTLs for rs1128175 and rs2853971 in the 2000HIV cohort. Only nominally significant associations are shown and all effect sizes are given with respect to the alternative allele.
(XLSX)

S2 Table Overview of HLA types of donors included in first intracellular cytokine staining experiment. (XLSX)

S3 Table Overview of HLA types and SNP genotypes of donors included in the second intracellular cytokine staining experiment. (XLSX)

S4 Table eQTL summary statistics for eQTLs for rs1128175 and rs2853971 from the 2000HIV cohort. Effect sizes are given with respect to the alternative allele.
(XLSX)

S5 Table Summary statistics of eQTLs from the GTEx Whole blood. Effect sizes are given with respect to the alternative allele.
(XLSX)

S6 Table Differential expression analysis of genes in a 1 Mb from rs1128175 and rs2853971. Differential expression analysis was carried out in 96 HICs versus 1363 non-HICs in the discovery cohort.
(XLSX)

S7 Table Summary statistics mQTL mapping for CpGs in a 1 Mb window from rs1128175 and rs2853971. (XLSX)

S8 Table Full caQTL mapping summary statistics of cis-chromatin accessibility features for rs1128175 and rs2853971. All effect sizes are given with respect to the A- alleles.
(XLSX)

S9 Table Cell-count QTL summary statistics for rs1128175 and rs2853971. Effect sizes are given with respect to the alternative allele.
(XLSX)
