# Supplementary figures and images for: Co-regulation of HIV control and cytomegalovirus pp65-specific IL-1β and TNF-α responses by genetic variants in the MHC region
Source: PLoS Pathog. 2026 Jul 14;22(7):e1014355. doi: 10.1371/journal.ppat.1014355 (PMC13367729; doi:10.1371/journal.ppat.1014355)

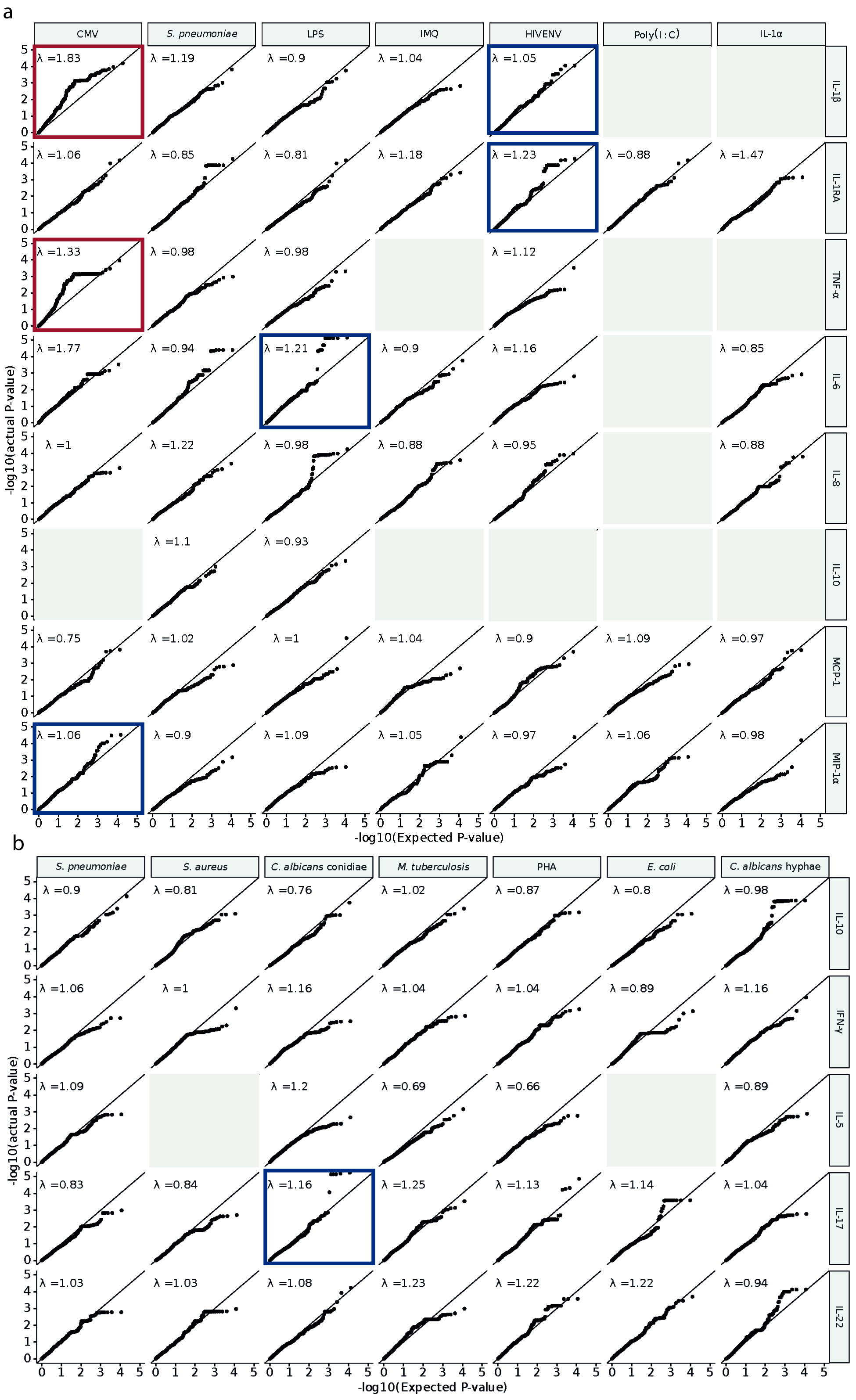

Supplement: S2 Fig — (a) Enrichment results for cQTLs for cytokine production measured after 24 hours of stimulation. (b) Enrichment results for cQTLs for cytokine production measured after 7 days of stimulation. Enrichment results are split per stimulus (horizontal) and cytokine (vertical), grey squares represent cytokine-stimuli pairs that were not measured. Shown are SNPs that pass the suggestive significance threshold in the cQTL mapping for the respective cytokine-stimulus pair. The x-axis shows the expected -log10(P-value) under a uniform distribution. The y-axis represents the actual -log10(P-value) in the GWAS for HIV control. Lambda values for genomic inflation are shown. Blue boxes indicate cytokine-stimuli pairs for which enrichment of HIV control associated SNPs was found in cQTLs at P < 1 ⋅ 10-3. Red boxes indicate cytokine-stimuli pairs for which consistent enrichment of HIV control associated SNPs in the cQTLs was found over 3 P-value thresholds. (TIF) [file ppat.1014355.s002.tif]

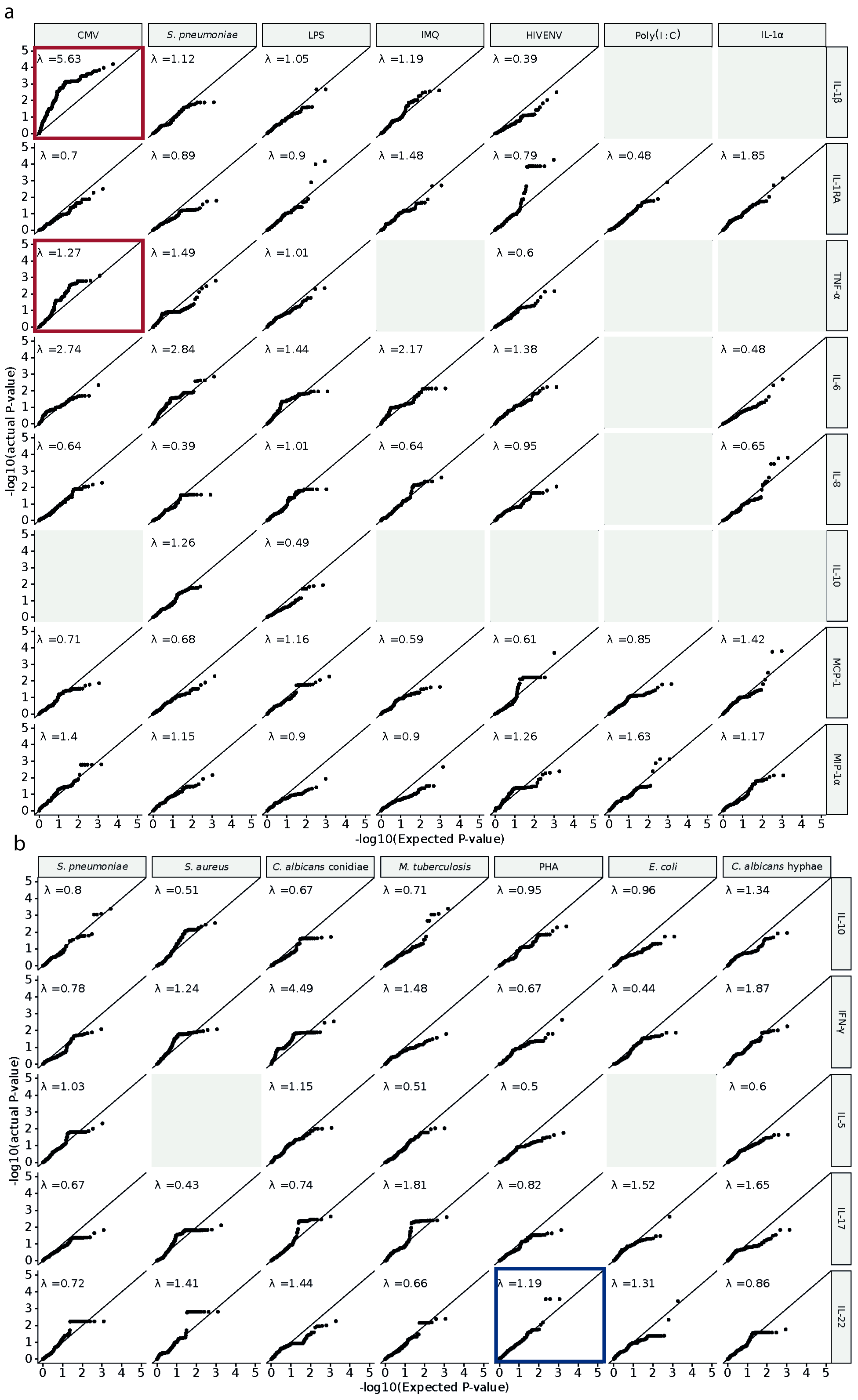

Supplement: S3 Fig — (a) Enrichment results for cQTLs for cQTLs for cytokine production measured after 24 hours of stimulation. (b) Enrichment results for cytokine production measured after 7 days of stimulation. Enrichment results are split per stimulus (horizontal) and cytokine (vertical), grey squares represent cytokine-stimuli pairs that were not measured. Shown are SNPs that pass the suggestive significance threshold in the cQTL mapping for the respective cytokine-stimulus pair. The x-axis shows the expected -log10(P-value) under a uniform distribution. The y-axis represents the actual -log10(P-value) in the GWAS for HIV control. Lambda values for genomic inflation are shown. Blue boxes indicate cytokine-stimuli pairs for which enrichment of HIV control associated SNPs was found in cQTLs at P < 1 ⋅ 10-4. Red boxes indicate cytokine-stimuli pairs for which consistent enrichment of HIV control associated SNPs in the cQTLs was found over 3 P-value thresholds. (TIF) [file ppat.1014355.s003.tif]

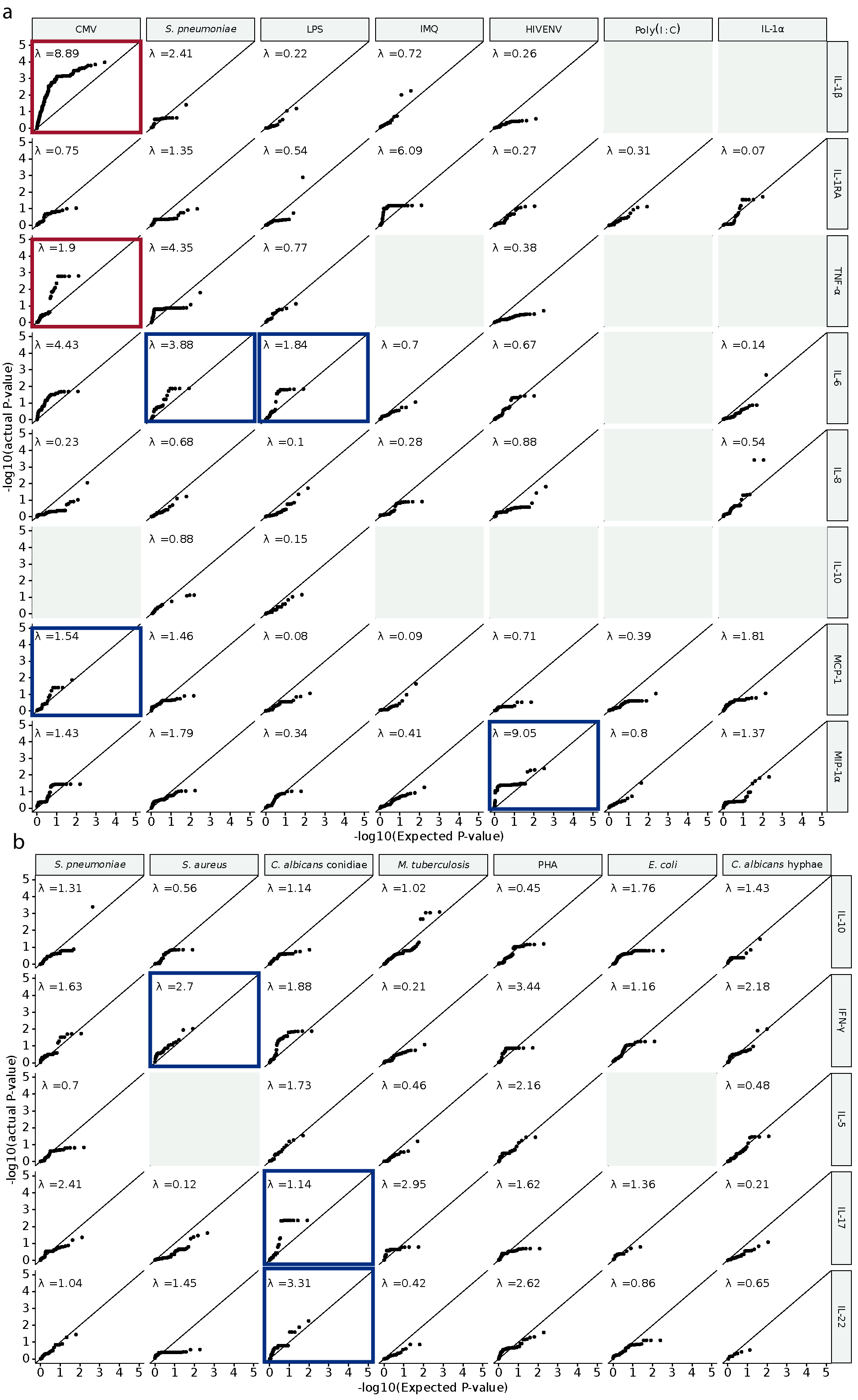

Supplement: S4 Fig — (a) Enrichment results for cQTLs for cytokine production measured after 24 hours of stimulation. (b) Enrichment results for cQTLs for cytokine production measured after 7 days of stimulation. Enrichment results are split per stimulus (horizontal) and cytokine (vertical), grey squares represent cytokine-stimuli pairs that were not measured. Shown are SNPs that pass the suggestive significance threshold in the cQTL mapping for the respective cytokine-stimulus pair. The x-axis shows the expected -log10(P-value) under a uniform distribution. The y-axis represents the actual -log10(P-value) in the GWAS for HIV control. Lambda values for genomic inflation are shown. Blue boxes indicate cytokine-stimuli pairs for which enrichment of HIV control associated SNPs was found in cQTLs at P < 1 ⋅ 10-5. Red boxes indicate cytokine-stimuli pairs for which consistent enrichment of HIV control associated SNPs in the cQTLs was found over 3 P-value thresholds. (TIF) [file ppat.1014355.s004.tif]

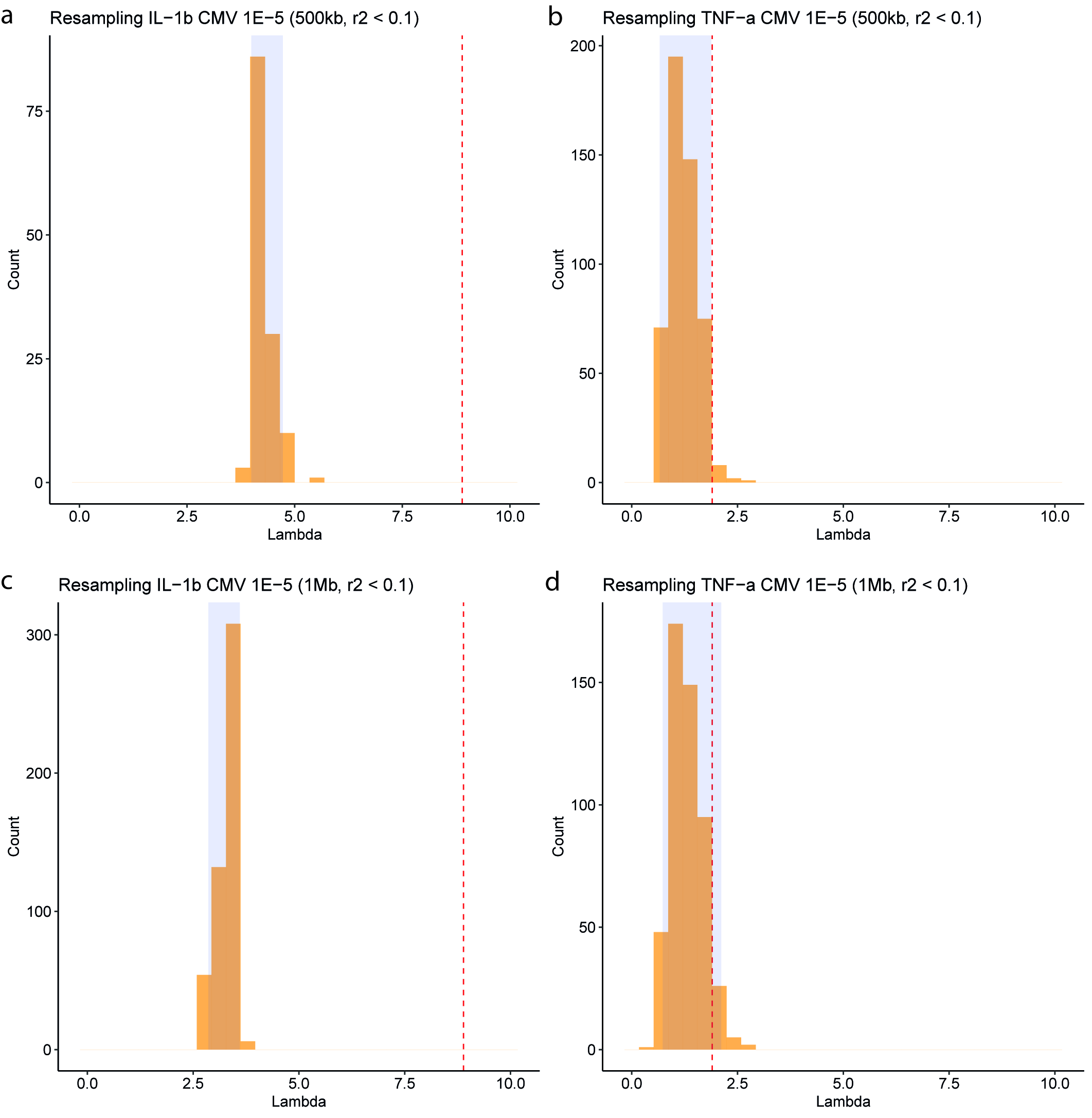

Supplement: S5 Fig — For each of the 500 permutations, for each cQTL that passed the suggestive threshold (P < 1 • 10-5), a random SNP was selected within 500kb (a,b) or 1 Mb (c,d) distance, with a similar MAF (+- 0.05), not in LD with the original SNP (r2 < 0.1). Using these randomly selected SNPs, the lambda value was recalculated. Yellow histograms represent the distributions of these lambdas, and the blue shading represents the 95% confidence interval. The red dashed line represents the lambda value in the original enrichment analysis. (TIF) [file ppat.1014355.s005.tif]

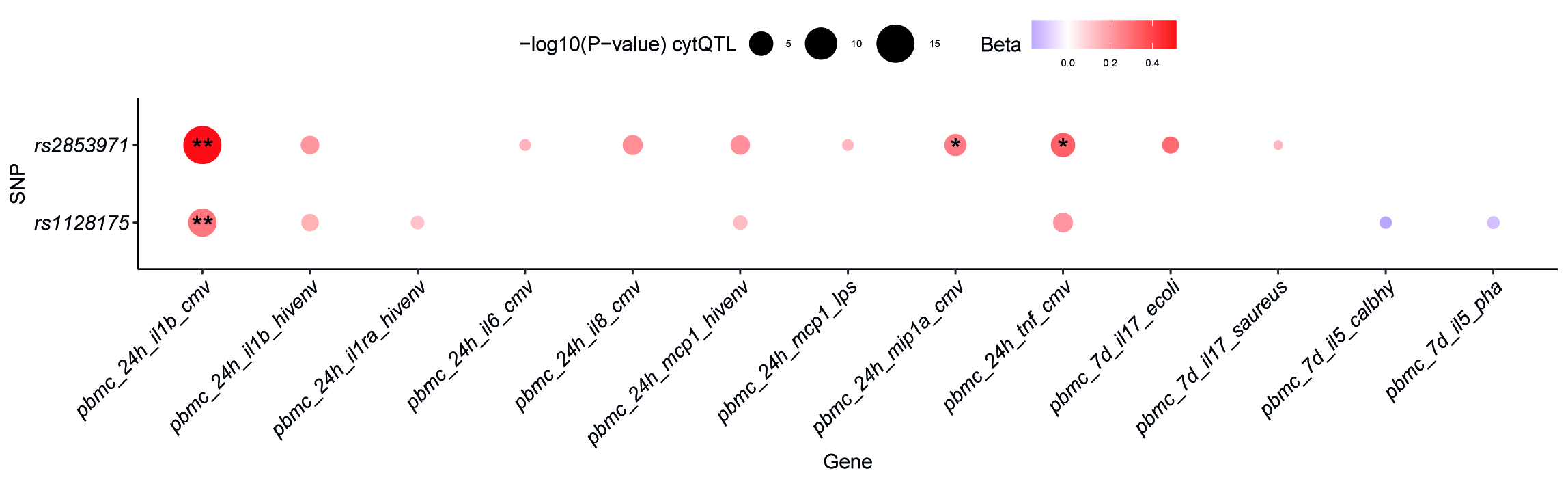

Supplement: S6 Fig — The x-axis shows all cytokine-stimuli pairs for which suggestive associations (Pdiscovery < 0.05) were found and the y-axis shows the two top enriched SNPs. The size of the dots indicates the -log10(P-value) in the discovery cohort and the color indicates the beta value with respect to the HIV control risk allele, with red indicating that the HIV control risk allele is associated with increased cytokine production. * indicates an effect (Pdiscovery < 3.2 • 10-4) ** indicates an effect (Pdiscovery < 3.2 • 10-4) validated in the validation cohort (Pvalidation < 0.05). (TIF) [file ppat.1014355.s006.tif]

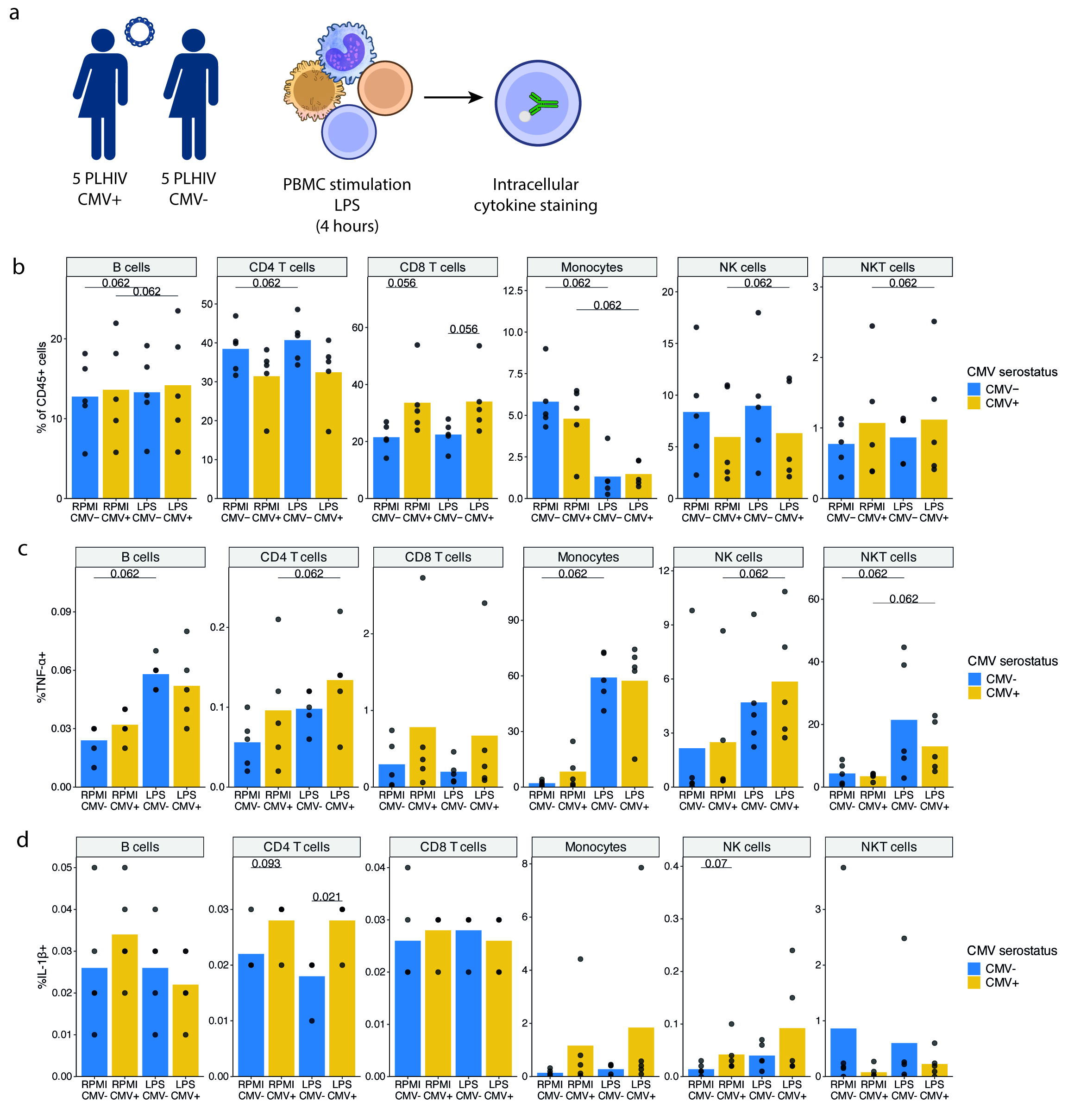

Supplement: S7 Fig — (a) Schematic overview of the experimental design. PBMCs from 10 PLHIV (5 CMV+ and 5 CMV-) were stimulated with RPMI (negative control) or LPS (positive control) for 4 hours, of which 3 hours in the presence of brefeldin A. The cellular composition and intracellular TNF-α and IL-1β expression in CMV+ and CMV- individuals were assessed using flow cytometry. (b) Immune cell subsets B cells (CD19+), CD4 T cells (CD3 + CD4 + CD8-), CD8 T cells (CD3 + CD4-CD8+), monocytes (CD14+), NK cells (CD3-CD56+), and NKT cells (CD3 + CD56 + CD8-) as percentage of total CD45 + cells measured. (c + d) Percentage of TNF-α+ and IL-1β+ cells within each cell subset, respectively. Bar heights represent mean percentages, and color represents CMV serostatus; CMV- in blue and CMV+ in yellow. P-values < 0.10 from Wilcoxon rank-sum test (for comparisons between CMV serostatus groups) and Wilcoxon signed-rank test (for comparisons within CMV serostatus groups) are shown. (TIF) [file ppat.1014355.s007.tif]

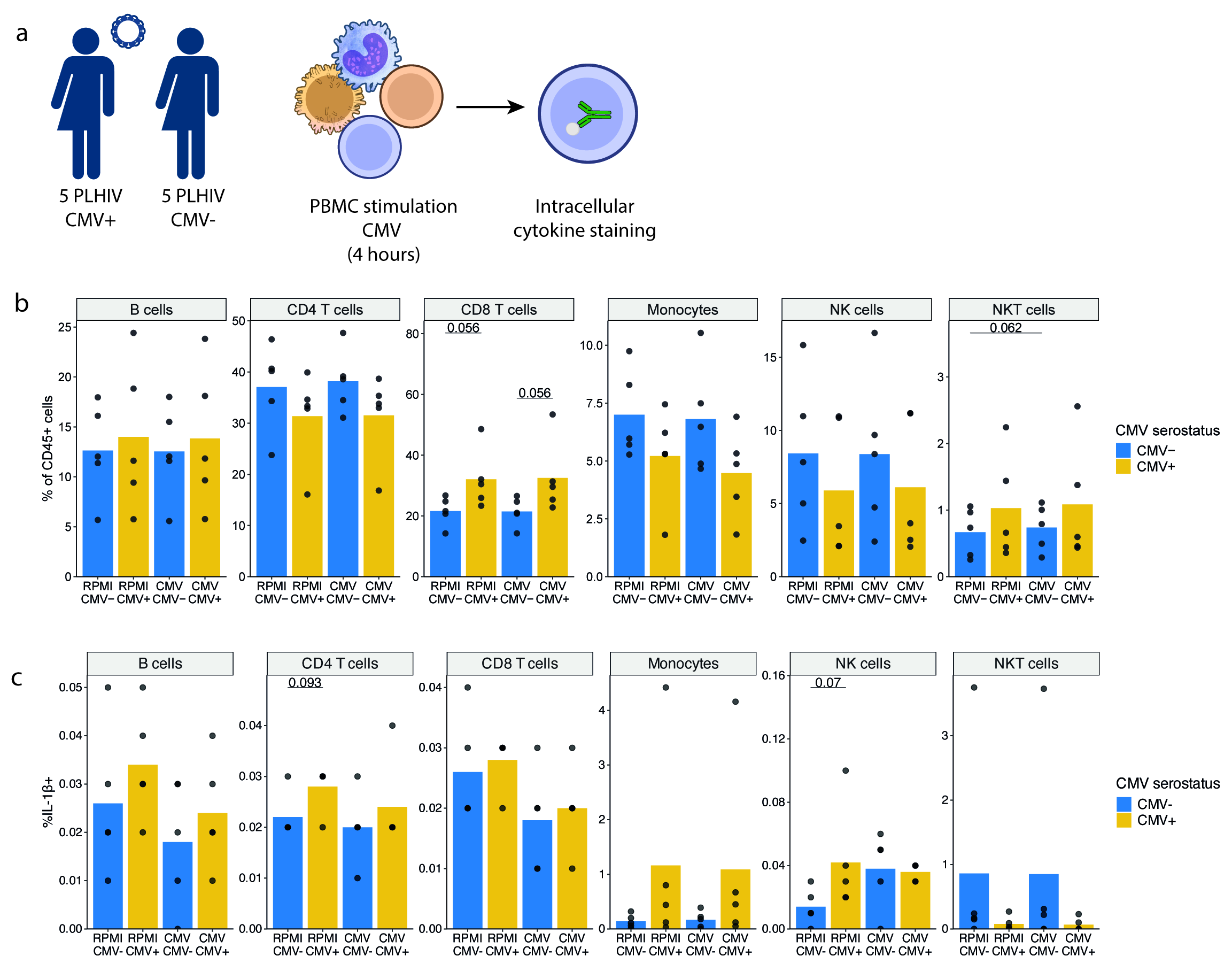

Supplement: S8 Fig — (a) Schematic overview of the experimental design. PBMCs from 10 PLHIV (5 CMV+ and 5 CMV-) were stimulated with RPMI (negative control) or a CMV pp65 peptide pool for 4 hours, of which 3 hours in the presence of brefeldin A. The cellular composition and intracellular IL-1β expression in CMV+ and CMV- individuals were assessed using flow cytometry. (b) Immune cell subsets B cells (CD19+), CD4 T cells (CD3 + CD4 + CD8-), CD8 T cells (CD3 + CD4-CD8+), monocytes (CD14+), NK cells (CD3-CD56+), and NKT cells (CD3 + CD56 + CD8-) as percentage of total CD45 + cells measured. (c) Percentage IL-1β+ cells within each cell subset. Bar heights represent mean percentages, and color represents CMV serostatus; CMV- in blue and CMV+ in yellow. P-values < 0.10 from Wilcoxon rank-sum test (for comparisons between CMV serostatus groups) and Wilcoxon signed-rank test (for comparisons within CMV serostatus groups) are shown. (TIF) [file ppat.1014355.s008.tif]

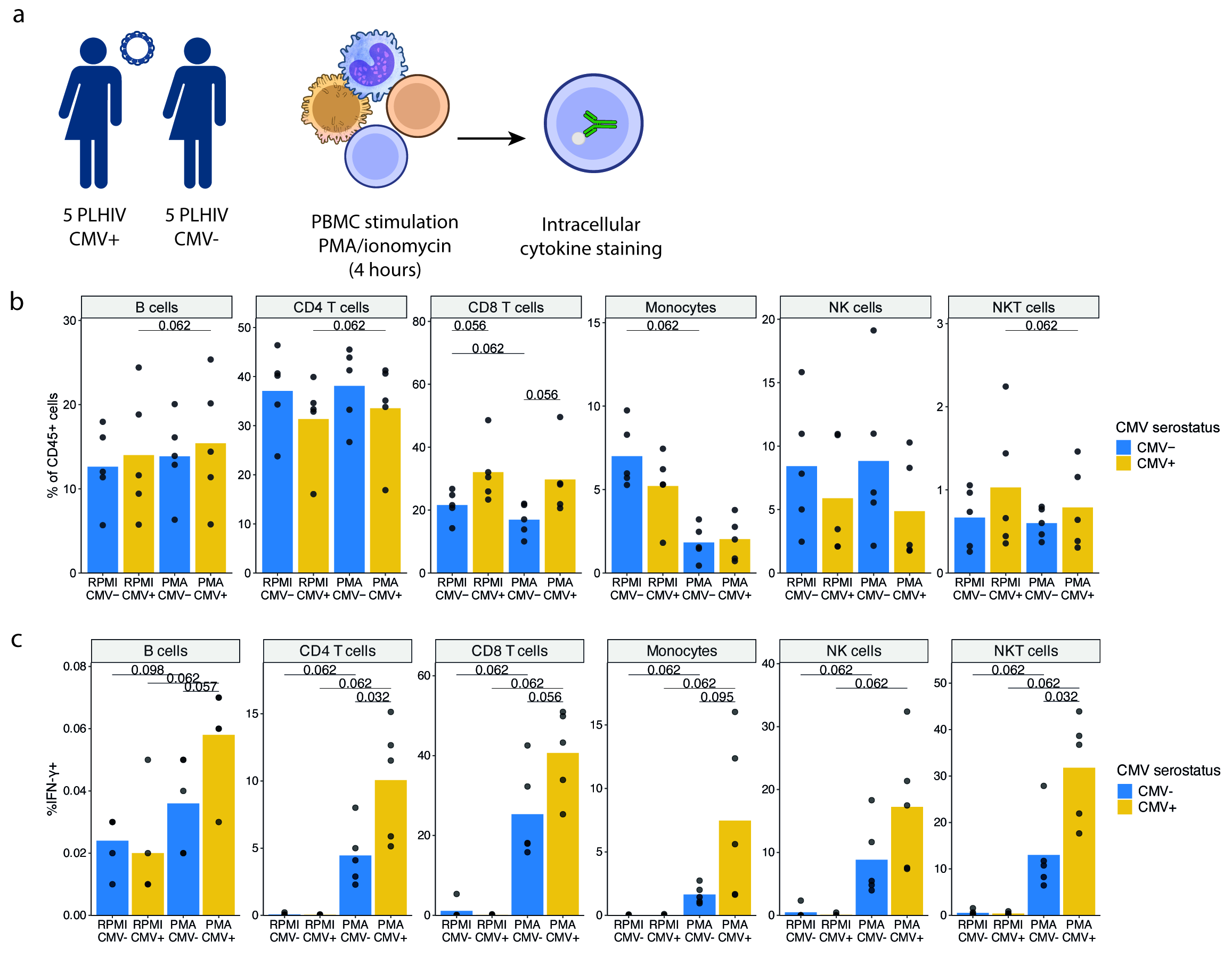

Supplement: S9 Fig — (a) Schematic overview of experimental procedure. PBMCs from 10 PLHIV (5 CMV+ and 5 CMV-) were stimulated with RPMI (negative control) or PMA/ionomycin (positive control) for 4 hours, of which 3 hours in the presence of brefeldin A. The cellular composition and intracellular IFN-γ expression in CMV+ and CMV- individuals were assessed using flow cytometry. (b) Immune cell subsets B cells (CD19+), CD4 T cells (CD3 + CD4 + CD8-), CD8 T cells (CD3 + CD4-CD8+), monocytes (CD14+), NK cells (CD3-CD56+), and NKT cells (CD3 + CD56 + CD8-) as percentage of total CD45 + cells measured. (c) Percentage of IFN-γ+ cells within each cell subset. Bar heights represent mean percentages, and color represents CMV serostatus; CMV- in blue and CMV+ in yellow. P-values < 0.10 from Wilcoxon rank-sum test (for comparisons between CMV serostatus groups) and Wilcoxon signed-rank test (for comparisons within CMV serostatus groups) are shown. (TIF) [file ppat.1014355.s009.tif]

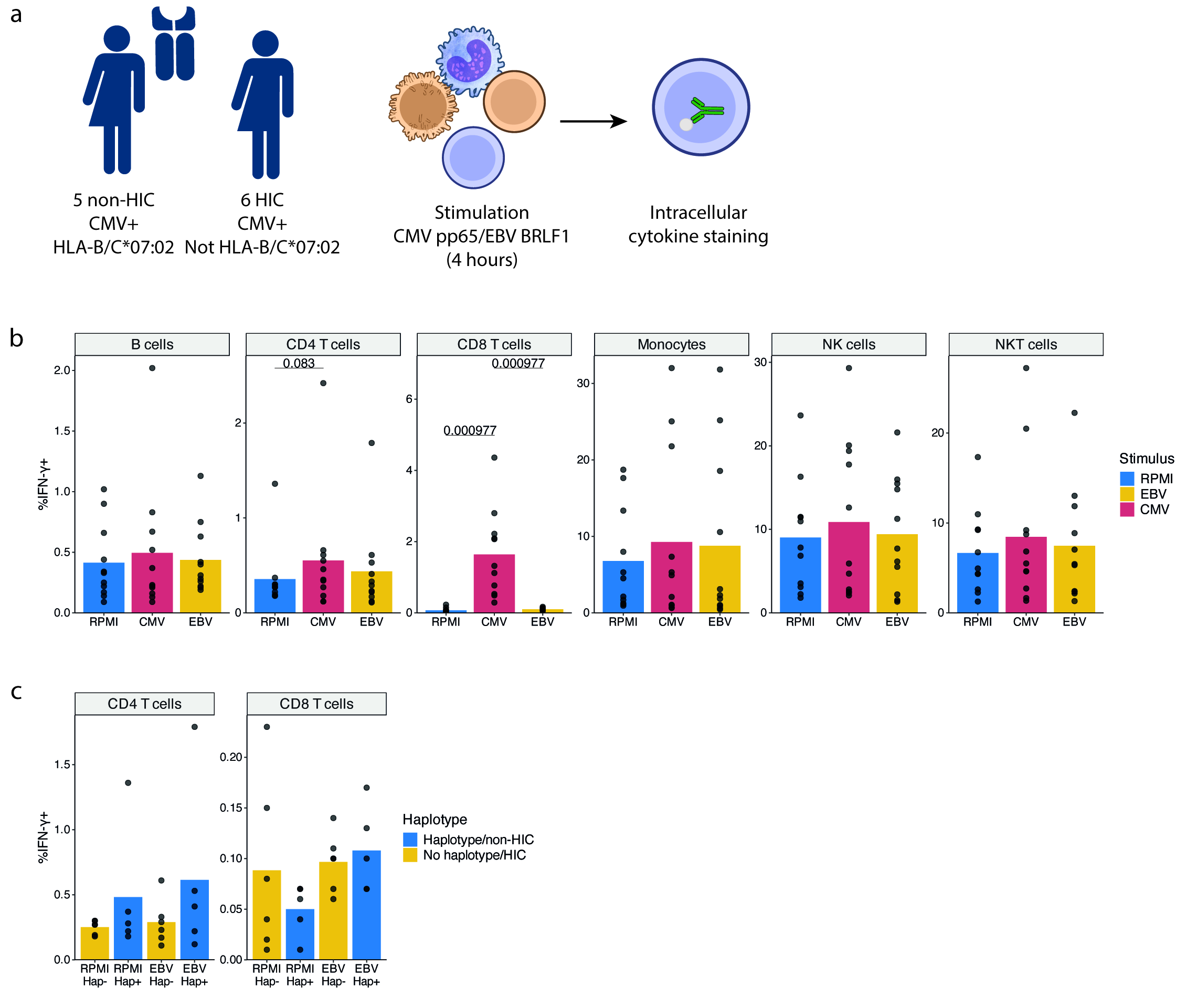

Supplement: S10 Fig — (a) Schematic overview of the experiment. PBMCs from 5 non-HICs carrying the rs1128175-A/rs2853971-A/HLA-B*07:02/HLA-C*07:02 haplotype (Hap+) and 6 HICs not carrying the haplotype (Hap-) were stimulated with a CMV pp65 or EBV BRLF1 peptide pool for 4 hours, of which 3 in the presence of brefeldin A. IFN-γ production was measured intracellularly by flow cytometry in six major cell populations: B cells (CD19+), CD4 T cells (CD3 + CD4 + CD8-), CD8 T cells (CD3 + CD4-CD8+), monocytes (CD14+), NK cells (CD3-CD56+), and NKT cells (CD3 + CD56 + CD8-). (b) Percentage of IFN-γ+ cells within each population (y-axis) stratified by stimulus (x-axis). Colors represent the stimulus. (c) Percentage of IFN-γ positive CD4 and CD8 T cells (y-axis) upon stimulation with EBV BRLF1 or RPMI as a control. Colors represent the presence of the haplotype. Statistical testing was performed using a Wilcoxon signed-rank test for comparisons within haplotype groups and a Wilcoxon rank-sum test for comparison between haplotype groups. Only P-values < 0.10 are shown. (TIF) [file ppat.1014355.s010.tif]

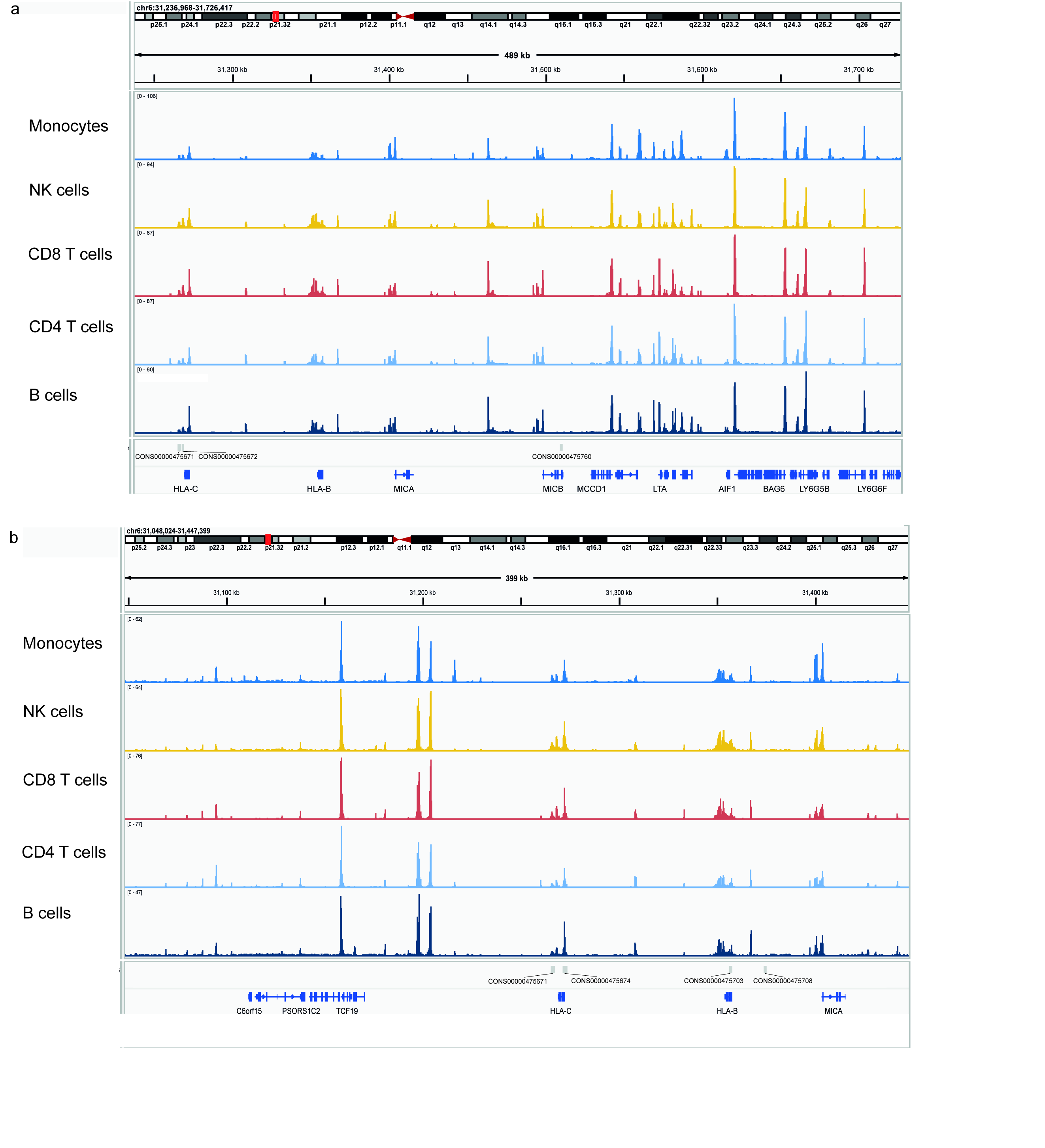

Supplement: S11 Fig — ATAC-seq counts are visualized for all individuals included in the ATAC-seq experiment per cell type. The main peaks for which caQTLs were found are labelled in the respective plot. (TIF) [file ppat.1014355.s011.tif]

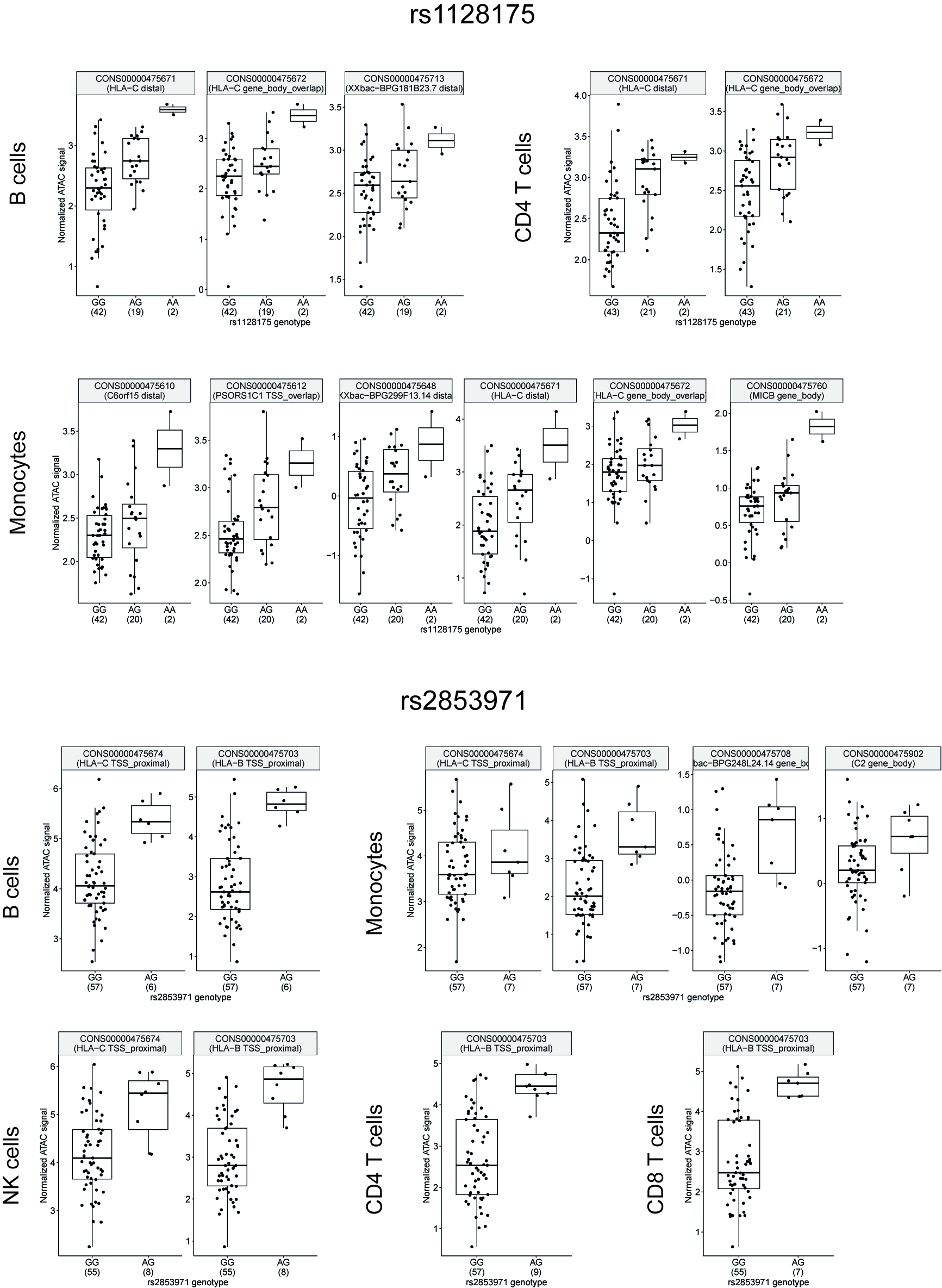

Supplement: S12 Fig — The x-axis shows the genotypes (count) and the y-axis the normalized chromatin ATAC sequencing signal. (TIF) [file ppat.1014355.s012.tif]

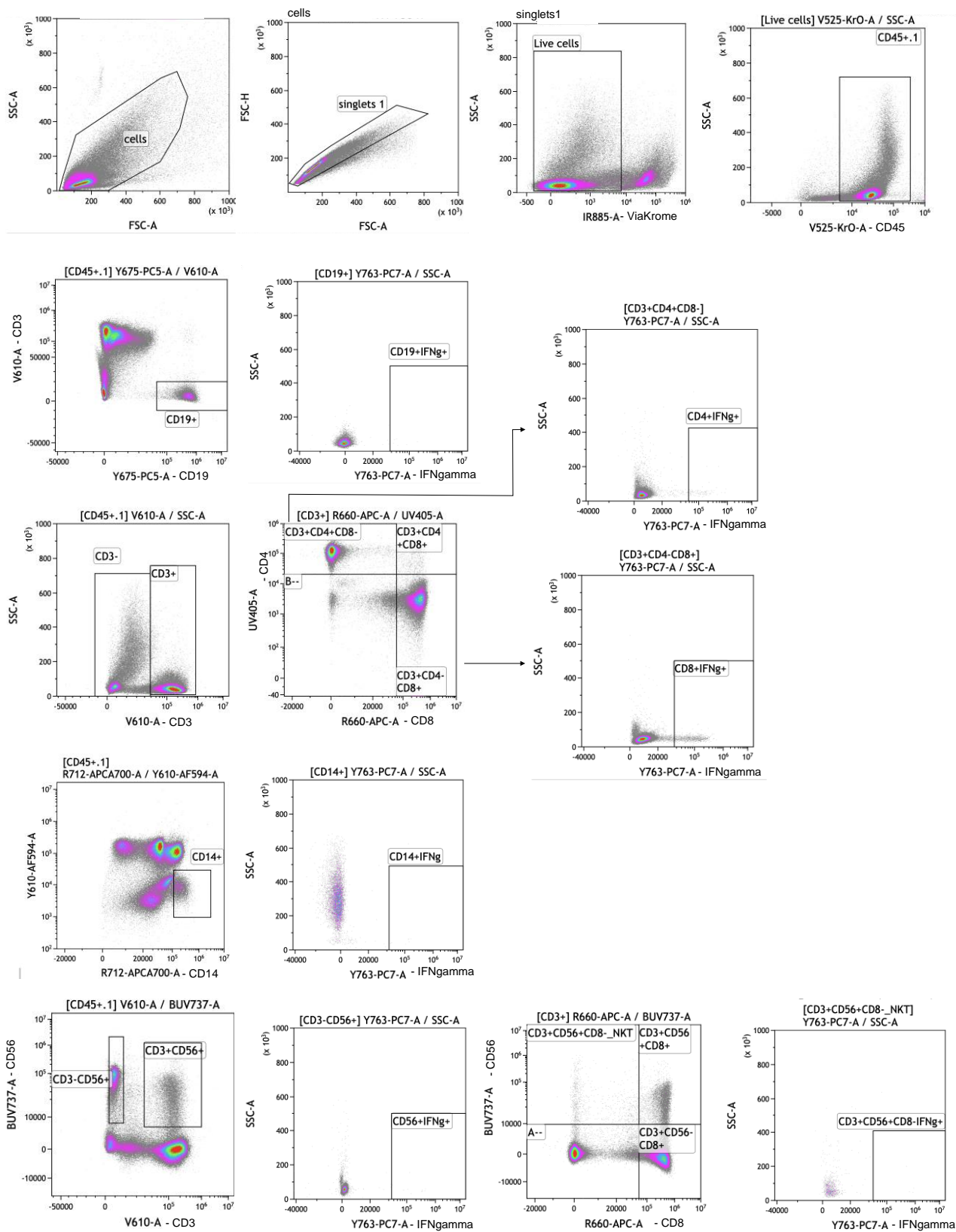

Supplement: S13 Fig — (PDF) [file ppat.1014355.s013.pdf]

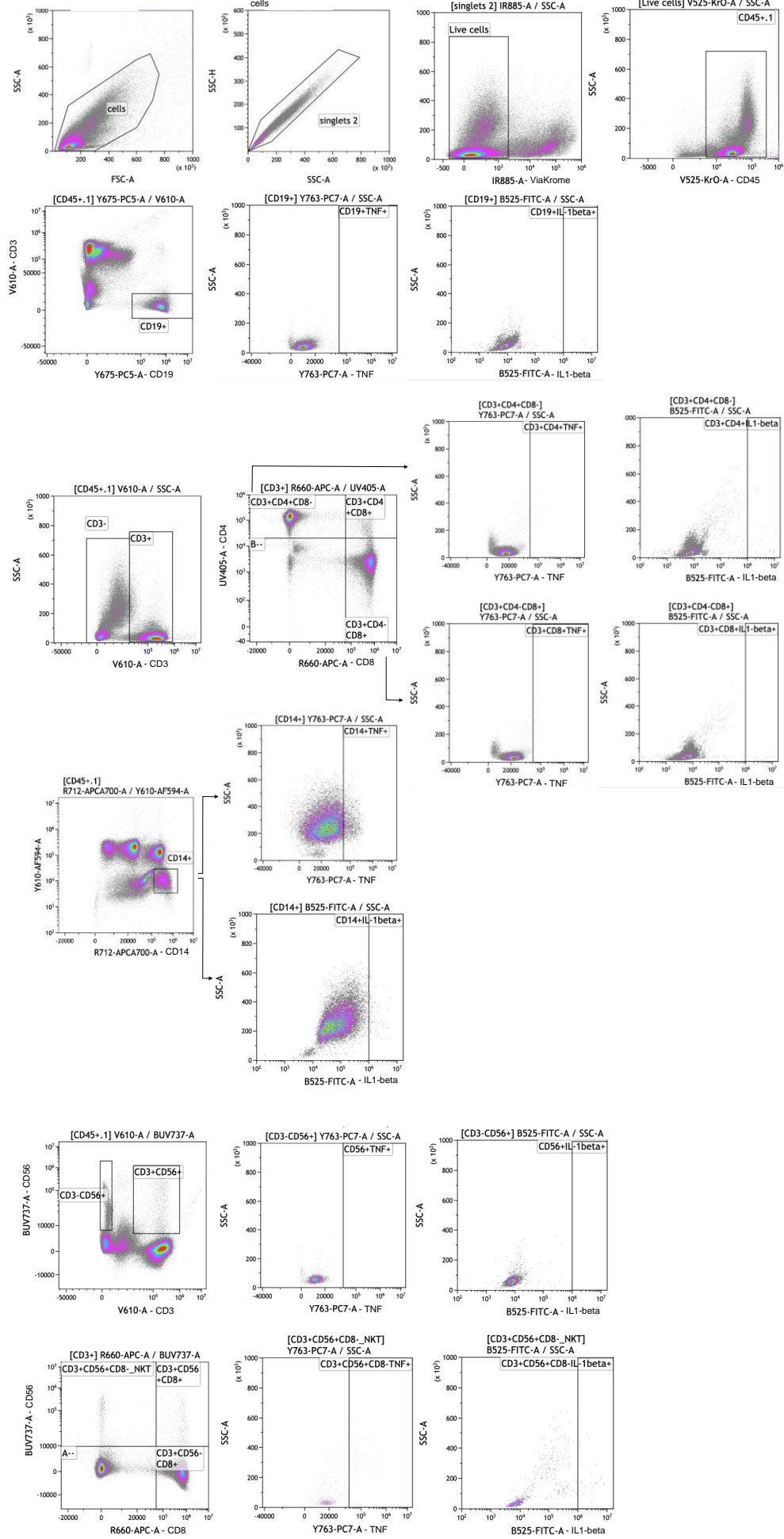

Supplement: S14 Fig — (PDF) [file ppat.1014355.s014.pdf]
